# Exploring the evolutionary route of the acquisition of betaine aldehyde dehydrogenase activity by plant ALDH10 enzymes: implications for the synthesis of the osmoprotectant glycine betaine

**DOI:** 10.1186/1471-2229-14-149

**Published:** 2014-05-29

**Authors:** Rosario A Muñoz-Clares, Héctor Riveros-Rosas, Georgina Garza-Ramos, Lilian González-Segura, Carlos Mújica-Jiménez, Adriana Julián-Sánchez

**Affiliations:** 1 Departamento de Bioquímica, Facultad de Química, Universidad Nacional Autónoma de México, México D.F., México; 2 Departamento de Bioquímica, Facultad de Medicina, Universidad Nacional Autónoma de México, México D.F., México

**Keywords:** Osmoprotection, Osmotic stress, Aminoaldehyde dehydrogenase, Enzyme kinetics, Substrate specificity, Enzyme subcellular location, Protein stability, Protein structure, Protein evolution

## Abstract

**Background:**

Plant ALDH10 enzymes are aminoaldehyde dehydrogenases (AMADHs) that oxidize different ω-amino or trimethylammonium aldehydes, but only some of them have betaine aldehyde dehydrogenase (BADH) activity and produce the osmoprotectant glycine betaine (GB). The latter enzymes possess alanine or cysteine at position 441 (numbering of the spinach enzyme, *So*BADH), while those ALDH10s that cannot oxidize betaine aldehyde (BAL) have isoleucine at this position. Only the plants that contain A441- or C441-type ALDH10 isoenzymes accumulate GB in response to osmotic stress. In this work we explored the evolutionary history of the acquisition of BAL specificity by plant ALDH10s.

**Results:**

We performed extensive phylogenetic analyses and constructed and characterized, kinetically and structurally, four *So*BADH variants that simulate the parsimonious intermediates in the evolutionary pathway from I441-type to A441- or C441-type enzymes. All mutants had a correct folding, average thermal stabilities and similar activity with aminopropionaldehyde, but whereas A441S and A441T exhibited significant activity with BAL, A441V and A441F did not. The kinetics of the mutants were consistent with their predicted structural features obtained by modeling, and confirmed the importance of position 441 for BAL specificity. The acquisition of BADH activity could have happened through any of these intermediates without detriment of the original function or protein stability. Phylogenetic studies showed that this event occurred independently several times during angiosperms evolution when an *ALDH10* gene duplicate changed the critical Ile residue for Ala or Cys in two consecutive single mutations. ALDH10 isoenzymes frequently group in two clades within a plant family: one includes peroxisomal I441-type, the other peroxisomal and non-peroxisomal I441-, A441- or C441-type. Interestingly, high GB-accumulators plants have non-peroxisomal A441- or C441-type isoenzymes, while low-GB accumulators have the peroxisomal C441-type, suggesting some limitations in the peroxisomal GB synthesis.

**Conclusion:**

Our findings shed light on the evolution of the synthesis of GB in plants, a metabolic trait of most ecological and physiological relevance for their tolerance to drought, hypersaline soils and cold. Together, our results are consistent with smooth evolutionary pathways for the acquisition of the BADH function from ancestral I441-type AMADHs, thus explaining the relatively high occurrence of this event.

## Background

The synthesis of the osmoprotectant glycine betaine (GB) is a metabolic trait of great adaptive importance that allows the plants possessing it to contend with osmotic stress caused by drought, salinity or low temperatures. Since these adverse environmental conditions are the major limitations of agricultural production, engineering the synthesis of GB in crops that naturally lack this ability has been, and still is, a biotechnological goal for improving their tolerance to osmotic stress (reviewed in [1]). Also, it is becoming increasingly appreciated that the GB content of an edible plant is valuable for human and animal nutrition [2].

In plants, GB is formed by the NAD^+^-dependent oxidation of betaine aldehyde (BAL) catalyzed by betaine aldehyde dehydrogenases (BADHs). Within the aldehyde dehydrogenase (ALDH) superfamily, plant BADHs belong to the family 10 [3] whose members are ω-aminoaldehyde dehydrogenases (AMADHs) that *in vitro* can oxidize small aldehydes possessing an ω-primary amine group, such as 3-aminopropionaldehyde (APAL) and 4-aminobutyraldehyde (ABAL) [4-12], a trimethylammonium group, such as 4-trimethylaminobutyraldehyde (TMABAL) [9,12], or a dimethylsulfonium group, such as 3-dimethylsulfoniopropionaldehyde [4,5]. *In vivo*, depending of the substrate used, these enzymes may participate in diverse biochemical processes, which range from the catabolism of polyamines to the synthesis of several osmoprotectants (alanine betaine, 4-aminobutyrate, carnitine or 3-dimethylsulfoniopropionate) in addition to GB. Although the biochemically characterized plant ALDH10s oxidize all the above-mentioned aldehydes, only some of them efficiently use BAL as substrate [9,13-18] and therefore can participate in the synthesis of GB. The difference in BAL specificity among the plant ALDH10s was puzzling given the high structural similarity between BAL and the other ω-aminoaldehydes, as well as between the plant ALDH10 enzymes. Recently, by means of X-ray crystallography, *in silico* model building, site-directed mutagenesis, and kinetic studies of the ALDH10 enzyme from spinach (*So*BADH), we found that only an amino acid residue at position 441 is critical for an ALDH10 enzyme being able to accept or reject BAL as a substrate [19]. This residue, located in the second sphere of interaction with the substrate behind the indole group of the tryptophan equivalent to W456 in *So*BADH, determines the size of the pocket formed by the Trp and Tyr residues equivalent to Y160 and W456 (*So*BADH numbering) where the bulky trimethylammonium group of BAL binds. If this residue is an Ile it pushes the Trp against the Tyr, thus hindering the binding of BAL, whereas if it is an Ala or a Cys the Trp adopts a conformation that leaves enough room for productive BAL binding [19]. This conclusion was drawn by Díaz-Sánchez *et al.*[19] by comparing the crystal structures of the *So*BADH (PDB code 4A0M) with those of the two pea AMADH enzymes, which do not have BADH activity (*Ps*AMADH1 and *Ps*AMADH2, PDB codes 3IWK and 3IWJ, respectively, [12]), and was later confirmed by Kopěcný *et al*. [20] when they reported the crystal structures of the maize ALDH10 isoenzyme, which contains Cys at position equivalent to 441 (*So*BADH numbering) and exhibits BADH activity (*Zm*AMADH1a; PDB code 4I8P), and of a tomato ALDH10 isoenzyme, which contains Ile at this position and is devoid of BADH activity (*Sl*AMADH1; PDB code 4I9B). Moreover, by correlating the reported level of BADH activity of ALDH10 enzymes with the presence of either of these residues, Díaz-Sánchez *et al.*[19] predicted that those enzymes that have an Ile at position 441—which we will name hereafter as I441-type isoenzymes—would have only AMADH activity while those that have either Ala or Cys—which we will name hereafter as A441- or C441-type isoenzymes—would exhibit also BADH activity. And since an almost perfect correlation was found between the reported ability of the plant to accumulate GB and the presence of an ALDH10 isoenzyme with proved or predicted BADH activity, it was proposed that the absence of this kind of isoenzyme is a major limitation for the synthesis of GB in plants [19]. Indeed, a significant BADH activity would be necessary not only to produce significant levels of GB but also to prevent the accumulation of BAL, which is formed in the oxidation of choline by choline monooxygenase (CMO), up to toxic concentrations.

Amino acid sequence analysis showed that most plants have two ALDH10 isoenzymes, probably as a consequence of gene duplication, and that the I441-type isoenzyme was the commonest [19]. The latter observation led to the suggestions that this residue corresponds to the ancestral feature in the plant ALDH10 family, and that a functional specialization occurred in some plants when the Ile at position equivalent to 441 of *So*BADH mutated to Ala or Cys in one of the two copies of the duplicated gene [19]. Since the codons for Ile differ from those for Ala or Cys in two positions, we reasoned that any of these changes had to occur through an intermediate. To explore the evolutionary history of the synthesis of GB in plants, we generated and characterized the *So*BADH mutants A441V, A441S, A441T and A441F, which simulate the four parsimonious intermediates in the pathway from the plant ALDH10 isoenzymes exhibiting only AMADH activity, exemplified by the A441I *So*BADH mutant, to those that also exhibited BADH activity, exemplified by the wild-type *So*BADH or the A441C mutant. In this work, by comparing the kinetic properties and the thermo-stabilities of the mutants with those of the wild-type enzyme, we confirm that the size of the residue at position 441 greatly affect the specificity for betaine aldehyde, and conclude that the acquisition of the new BADH function occurred without detriment of either the oxidation of other aminoaldehydes or the protein stability. Also, we present here strong phylogenetic evidence that confirms that peroxisomal I441-type isoenzymes correspond to the ALDH10 ancestral form and that independent duplication events occurred in monocots and eudicots plants. Indeed, the change to A441-type isoenzymes was the commonest in eudicots, whereas the change to C441-type isoenzymes was in monocots.

## Results

### Phylogenetic analysis of the ALDH10 enzymes

**Figure 1:**
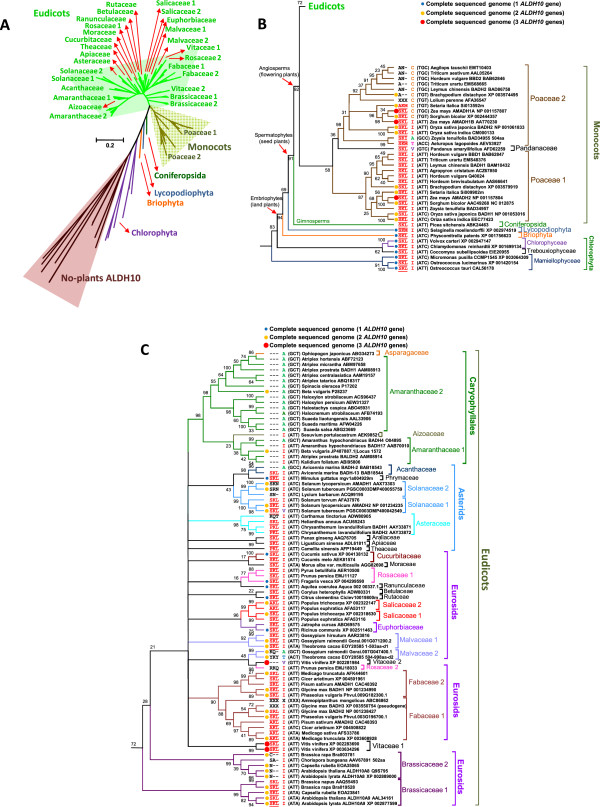
**Phylogenetic analysis of plant ALDH10 enzymes. A)** Unrooted phylogenetic tree that includes all identified ALDH10 protein sequences showing the taxonomic group to which they belong. **B)** Monocot and non-flowering plant ALDH10 sequences. **C)** Eudicot ALDH10 sequences. Indicated are the presence/absence of a peroxisomal-targeting signal PST1 that fits to the consensus sequence (S/A/C)-(K/R/H)-(L/M) (in red and underlined) as well as the amino acid residue and codon at position equivalent to 441 of *So*BADH. The tree was inferred from 500 replicates using the ML method [61]. The best tree with the highest log likelihood (-32886.4851) is shown. Similar trees were obtained with MP, ME and NJ methods. The analysis involved 131 amino acid sequences (122 from plants and 9 from non-plants). In panel A the branches of the unrooted tree are drawn to scale, with the bar length indicating the number of substitutions per site. In panels **B** and **C** only the branch topology is shown. The proportion of replicate trees in which the associated taxa clustered together in a bootstrap test (500 replicates) is given next to the branches. Branches with a very low bootstrap value (<20%) are collapsed. For each sequence, the accession number and the name assigned in published papers (in the case of proteins previously studied) are given. X indicates an unidentified amino acid/nucleotide.

We expanded the amino acid sequence alignments of plant ALDH10 enzymes, including in this phylogenetic study three times more sequences than in previous works [19,20]. The retrieved non-redundant sequences belong mainly to plants (122 sequences), but ALDH10 proteins were also found in fungi, protists, and proteobacteria; none in animals or archea (Additional file 1: Table S1). Figure 1 shows an unrooted phylogenetic tree that includes all identified ALDH10 sequences (panel A), as well as detailed phylogenetic trees from monocots (panel B) and eudicots (panel C). As expected, land plants (Embriophytes) form a well-supported monophyletic group, as well as Spermatophytes (seed plants) and Angiosperms (flowering plants). In Figure 1B it can be observed that primitive plants with a known genome like *Ostrococcus tauri, O. lucimarinus, Micromonas pusilla, Chlamydomonas reinhardtii, Volvox carteri* (Chlorophyta), *Physcomitrella patents* (Briophyta) and *Selaginella moellendorffii* (Lycopodiophyta) contain only one ALDH10 enzyme. Interestingly, all these enzymes possess Ile at position equivalent to 441 (*So*BADH numbering), which is also the residue most frequently found in ALDH10 enzymes of the other plant families (Figures 1B and 1C). Thus, among the 122 non-redundant plant ALDH10 sequences analyzed, 88 possess Ile, 19 Ala, and 10 Cys. Only three ALDH10 isoenzymes—from *Vitis vinifera, Solanum tuberosum* and *Pandanus amaryllifolius*— have Val at this position, and two—from *Auluropus lagopoides* and *Theobroma cacao*—have Thr. These data strongly support the previous proposal [19] that I441-type isoenzymes correspond to the ancestral protein of the ALDH10 family.

Figure 1B also shows that all known monocot *ALDH10* genes cluster together, which suggests that the duplicated *ALDH10* genes in monocots originated after the monocot-eudicot divergence. All monocot plants of known genome possess two genes coding for ALDH10 proteins, except maize that possesses three genes. As previously found [20], in the Poaceae family—which includes most of the known sequences from monocots—each of the two *ALDH10* genes forms a different clade in the phylogenetic tree: one (which we name Poaceae 1) exclusively includes I441-type isoenzymes while the second (which we name Poaceae 2) mainly contains C441-type. Because the limited number of monocot ALDH10 sequences available, it is not yet possible to know whether or not every monocot family, besides Poaceae, possess two ALDH10 isoenzymes.

Eudicots of known genomes have a variable number of genes coding for ALDH10 proteins (Figure 1C). Some species have only one gene—*Ricinus communis* (Euphorbiaceae), *Citrus clementina* (Rutaceae), *Aquilegia coerulea* (Ranunculaceae), *Fragaria vesca* (Rosaceae), *Cucumis sativus* (Cucurbitaceae), and *Mimulus guttatus* (Phrymaceae)—, others two genes—*Arabidopsis thaliana, A. lyrata, Capsella rubella, Brassica rapa* (Brassicaceae), *Glycine max*, *Medicago truncatula* (Fabaceae), *Gossypium raimondii, Theobroma cacao* (Malvaceae), *Populus trichocarpa* (Salicaceae), *Solanum lycopersicum, S. tuberosum* (Solanaceae), and *Beta vulgaris* (Amaranthaceae)—, and another—*Vitis vinifera* (Vitaceae)—three genes. *Glycine max,* in addition to the two *ALDH10* genes, possesses an additional copy that corresponds to a pseudogene. The complex distribution pattern exhibited by *ALDH10* genes in eudicots strongly suggests that several independent gene-duplication events occurred during their evolution after monocot-eudicot divergence (Figure 1C). Thus, at least four independent duplication events, those that took place in Fabaceae, Salicaceae, Solanaceae and Amaranthaceae, exhibit a very high bootstrap support (>90%). In species of the Brassicaceae, Fabaceae, Salicaceae, Rosaceae, and Solanaceae families the protein coded by the duplicate gene conserved the Ile at the position equivalent to 441, but in plants of the Amaranthaceae and Acanthaceae this residue was changed to an Ala, in Malvaceae to an Ala or Thr, and in the only sequenced species of Vitaceae to a Val. As in the case of monocots, two different clades can be observed in the phylogenetic tree of several eudicot families: the first includes the original I441-type isoenzymes, with the only exception of *Solanum tuberosum* where this Ile mutated to a Val; the second includes the duplicate I441-type isoenzymes or the A441-, V441- or T441-type derived from the I441-type. Interestingly, the majority of the A441-type isoenzymes are clustered in the Amaranthaceae 2 clade, with the exception of the A441-type of *Amaranthus hypochondriacus*, which is phylogenetically very close to the I441-type of the same plant, suggesting a recent duplication event. The genome of this plant has not been yet completely sequenced, so it could be that this plant possesses another A441-type isoenzyme that groups with the Amaranthaceae 2. Also, we cannot yet explain the unexpected position of the A441-type isoenzyme from *Ophiopogon japonicus* (a monocot), which clustered with the A441-type isoenzymes in the Amaranthaceae 2 clade. Since the A441-type isoenzymes are predicted to have BADH activity, i.e. the ability to oxidize BAL [19], it is interesting that *O. japonicus* CMO also has higher amino acid sequence identity with CMO proteins from the Amaranthaceae family than with CMO from monocots [21]. One possible explanation to this anomalous behavior is that both genes were acquired by *O. japonicus* by horizontal gene transfer, which is a significant force in the evolution of plant genomes [22,23]. Further studies are needed to provide evidence in favor or against this possibility.

We confirmed the previous observation [19,20] that the majority of the I441-type isoenzymes possesses a peroxisomal targeting signal type 1 (PST1) that fits to the consensus sequence (S/A)-(K/R)-(L/M/I) [24,25] whereas all the A441-type and the majority of C441-type isoenzymes lacks it (Figure 1). In the case of the C441-type the exceptions are those from maize, shorghum and foxtail millet (*Setaria italica*), which have the SKL signal and that of *Zoysia*, which have an SKI signal. The peroxisomal targeting signal was lost by the change of a residue or by truncation of the C-terminal region. The codon that encodes the missing Leu in the peroxisomal targeting signal of some of the C441-type isoenzymes of Poaceae 2 can be changed with only one punctual mutation to a stop codon. The same occurs with the genes that code for A441-type isoenzymes from the Amaranthaceae family, where the codons for the first missing Ser can be transformed with just one punctual mutation to a stop codon. The sequence divergence pattern of the Amaranthaceae supports the proposal that the loss of the peroxisomal signal PST1 occurred in this family before the gene duplication that gave rise to the A441-type isoenzymes. In other eudicot and monocot families, the enzymes with a truncated or mutated C-terminus cluster in the phylogenetic clades 2, a finding that gives additional support to the idea that these enzymes derived from the peroxisomal I441-type of clades 1.

### Possible ALDH10 evolutionary intermediates

**Figure 2:**
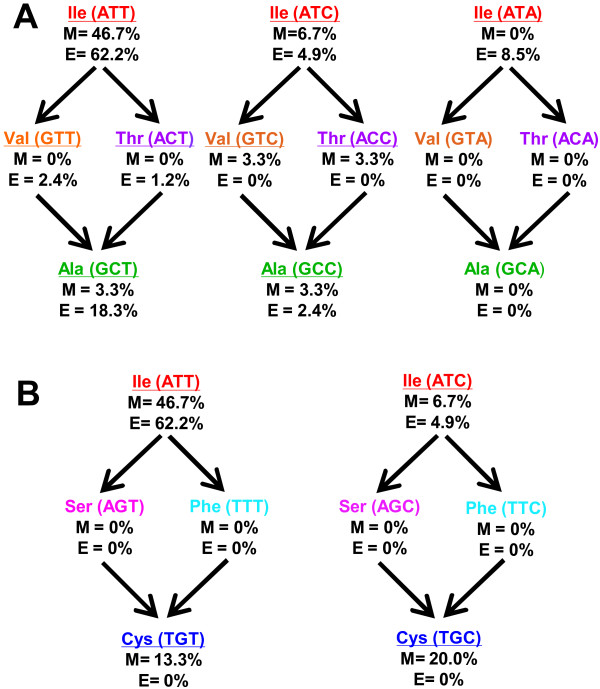
**Evolutionary pathways for the acquisition of BADH activity by plant ALDH10 enzymes.** Possible nucleotide changes in the pathway from the isoenzymes containing Ile at position 441 (*So*BADH numbering) to those containing Ala **(A)** or Cys **(B)** at this position. The frequency of the observed codons for monocots (M; 30 sequences), and eudicots (E; 82 sequences) is given. Experimentally observed codons and amino acids are underlined.

The phylogenetic analysis described above strongly supports that A441- and C441-type isoenzymes evolved from the I441-type ones. Ile can be coded by three different triplets, ATT, ATC and ATA, and of these the most frequently found in monocots and eudicots *ALDH10* genes is ATT and the least frequent ATA, which indeed was not found in monocots; alanine is coded by four, GCT, GCC, GCA, and GCG, of which GCT is the most used in eudicot *ALDH10* genes; and cysteine is coded by two, TGT and TGC, and both are present in monocots *ALDH10* genes. The observed frequency of each of these codons in monocots and eudicots is given in Figure 2A. From these data it can be observed that the triplet ATT at this position is more frequent than the ATC one.Since the most parsimonious pathways from Ile to either Ala or Cys involve two nucleotide substitutions, there should have been intermediates in the evolution from the I441-type to the A441- or C441-type ALDH10 isoenzymes. Several pathways could be followed depending on the Ile codon of the original enzyme, but in all cases the amino acid substitution in the evolutive intermediate has to be either Val or Thr in the pathway from Ile to Ala, and Phe or Ser in that from Ile to Cys (Figure 2B). This is consistent with the five different amino acids found at the critical position 441 in the ALDH10 enzymes of known sequence (Figure 1). Ile is the most frequently coded amino acid (71.9%), followed by Ala (15.7%), Cys (8.3%), Val (2.5%), and Thr (1.6%). Interestingly, ALDH10 isoenzymes containing Ser or Phe at position equivalent to 441, which are the possible intermediates in the pathway from I441 to C441, have not been found so far. Since C441-type isoenzymes are present in monocots, and the number of available ALDH10 sequences from monocots is still low (30 sequences) when compared with the number of available eudicot sequences (82 sequences), it is to be expected that the missing intermediates will be found when the number of known monocots ALDH10 sequences increases.

### Construction and kinetic characterization of the *So*BADH A441 mutants

To simulate the possible evolutionary intermediates we generated four *So*BADH variants: A441V, A441S, A441T, and A441F, and we characterized them, both kinetically and structurally. In a previous work [19] we had constructed the A441I mutant, which represents the putative original ALDH10 isoenzyme with only AMADH activity, i.e., devoid of significant BADH activity, and the A441C mutant, which, together with the wild-type *So*BADH, represents those isoenzymes that in addition to the AMADH activity also have BADH activity. The kinetics of the wild-type *So*BADH and of the A441I and A441C mutant enzymes were previously studied using BAL, APAL, ABAL and TMABAL as substrates [19], at pH 8.0 and at fixed 0.2 mM NAD^+^, conditions both that are nearly physiological [26,27]. Now we extend that work by studying the steady-state kinetics of the other four mutants with BAL or APAL as substrates. APAL can be used as representative of the other ω-aminoaldehydes that do not have a bulky trimethylammonium group close to the carbonyl group, and therefore their binding is not sterically constrained by the size of the cavity formed by the residues equivalent to Y160 and W456 (*Pa*BADH numbering). Consequently, the kinetics of these aldehydes are similar and very different from the kinetics of BAL, as previously found not only in the wild-type *So*BADH but also in the A441C and A441I mutants [19].

**Figure 3:**
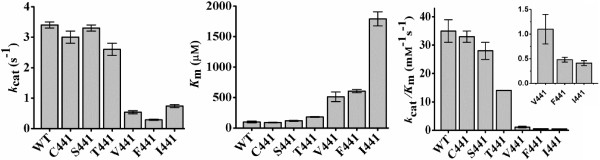
**Effects of mutation of A441 on the steady-state kinetic parameters of *So*BADH.** Wild-type and mutant *So*BADH enzymes were assayed at pH 8.0 and 30°C with BAL as variable substrate at fixed 0.2 mM NAD^+^. Other conditions are given in the Methods section. The kinetic parameter values were calculated from the best fit of initial velocity data to the Michaelis-Menten equation by non-linear regression. Each saturation curve was determined at least in duplicate using enzymes from two different purification batches. Bars indicate standard deviations. In the inset the *k*_cat_/*K*_m_ values of A441V, A441F and A441I are plotted using a scale smaller than that of the main figure.

Taking the kinetics of BAL as the criterion, two groups of enzymes were observed: one that includes the wild-type and the A441C, A441S and A441T mutants, which had a relatively high *k*_cat_, low *K*_m_(BAL) and high *k*_cat_/*K*_m_(BAL) values, and another formed by the A441V, A441F and A441I mutants, which exhibited low *k*_cat_, high *K*_m_(BAL), and very low *k*_cat_/*K*_m_(BAL), particularly the A441I mutant (Figure 3 and Table 1). The enzymes in the first group have, therefore, significant BADH activity, while the enzymes in the second group will be devoid of this activity at the expected intracellular BAL levels, which should not be high given the known toxicity of this aldehyde [28]. It has to be noted that the BADH activity of the enzymes in the first group is achieved not only by their much smaller *K*_m_(BAL) values, which most likely reflect a much better binding of the aldehyde, but also, although not so importantly, by their significantly higher *k*_cat_(BAL) values when compared with the enzymes of the second group. We also found clear differences between these two groups in their *K*_m_(NAD^+^) values, which were higher in the enzymes exhibiting BADH activity than in the enzymes with only AMADH activity, with the exception of the A441F (Table 1). As the *K*_m_ value for the first substrate in a bi-bi ordered steady-state kinetic mechanism depends not only on the second-order rate constant of the binding of this substrate but also in first-order rate constants associated with the steps after the central ternary complex is formed, the finding that the BADHs enzymes have higher *K*_m_(NAD^+^) values than those of the only-AMADHs ones could be due to the higher *k*_cat_ values of the former.

**Table 1:** **Steady-state kinetic parameters of wild-type and mutant *So*** **BADH enzymes in the oxidation of BAL**

		**Kinetic parameters**		
**Enzyme**	**Variable substrate**	***k***_***cat ***_**(s**^**-1**^**)**	***K***_**m **_**(μM)**	***k***_***cat***_ ***/K***_**m **_**(mM**^**-1**^**s**^**-1**^**)**
	**BAL**			
Wild type		3.36 ± 0.13	98 ± 15	35 ± 4
A441C		2.99 ± 0.19	90 ± 6	33 ± 2
A441S		3.29 ± 0.12	119 ± 8	28 ± 3
A441T		2.64 ± 0.16	180 ± 6	15 ± 1
A441V		0.54 ± 0.05	512 ± 79	1.1 ± 0.3
A441F		0.29 ± 0.02	605 ± 26	0.48 ± 0.05
A441I		0.74 ± 0.05	1791 ± 115	0.41 ± 0.05
	**NAD**^**+**^			
Wild type		4.25 ± 0.16	22 ± 2	195 ± 8
A441C		2.20 ± 0.09	14 ± 1	179 ± 17
A441S		3.27 ± 0.18	29 ± 3	114 ± 20
A441T		2.39 ± 0.00	24 ± 4	100 ± 15
A441V		0.51 ± 0.00	6.4 ± 0.5	80 ± 5
A441F		0.39 ± 0.02	18 ± 1	22 ± 0
A441I		0.68 ± 0.03	2.8 ± 0.0	243 ± 11

Initial velocities were obtained at 30°C in 50 mM HEPES-KOH buffer, pH 8.0, containing 0.1 mM EDTA. In the experiments with variable BAL, the fixed concentration of NAD^+^ was 0.2 mM, and in the experiments with variable NAD^+^ the fixed BAL concentrations were at least 10-times their app*K*_m_ values estimated for each enzyme at fixed 0.2 mM NAD^+^. The apparent kinetic parameters were estimated by non-linear regression fit of the experimental data to the Michaelis-Menten equation. The values given in the Table are the mean ± standard deviation of the kinetic parameters estimated in two duplicate saturation experiments performed with enzymes from two different purification batches. Values for *k*_*cat*_ are expressed per subunit.

The differences between the two groups of enzymes vanish when their kinetics with APAL as substrate are compared (Table 2), indicating that APAL oxidation was hardly affected by the kind of residue at position 441. The exception was the A441F mutant, which showed lower *k*_cat_/*K*_m_ values for both BAL and APAL than the wild-type and the other mutant enzymes, which suggest important structural alterations in the active site of this mutant. The catalytic efficiencies (*k*_cat_/*K*_m_) for APAL of the wild-type and mutants *So*BADH enzymes are higher than that for BAL (Table 2), as it has been found with other A441- or C441-type ALDH10 isoenzymes that in addition to the AMADH activity exhibit BADH activity [7,9,20]. Another difference between the kinetics with BAL and APAL is that APAL produced a small but clear substrate inhibition in the wild-type and mutant *So*BADHs, while this inhibition was not observed in the saturation kinetics with BAL in the concentration range studied. The observed degree of inhibition by high APAL concentrations was roughly the same in all the enzymes, but the highest APAL concentration used in our experiments, 0.2 mM, did not allow the accurate estimation of the substrate inhibition constant, K_IS_, values, which are not given in Table 2 for this reason. As previously shown [29], substrate inhibition arises from the non-productive binding of the aldehyde to the enzyme-NADH complex. Therefore, these results suggest that the enzyme-NADH complexes have lower affinity for BAL than for APAL, as it also the case of the productive enzyme-NAD^+^ complexes, as judged by the *K*_m_ values for the aldehydes.

**Table 2:** **Steady-state kinetic parameters of wild-type and mutant *So*** **BADH enzymes in the oxidation of APAL**

		**Kinetic parameters**		
**Enzyme**	**Variable substrate**	***k***_***cat ***_**(s**^**-1**^**)**	***K***_**m **_**(μM)**	***k***_***cat***_ ***/K***_**m **_**(mM**^**-1**^**s**^**-1**^**)**
	**APAL**			
Wild type		0.99 ± 0.04	3.9 ± 0.1	256 ± 5
A441C		0.67 ± 0.02	0.72 ± 0.16	931 ± 188
A441S		1.12 ± 0.00	2.0 ± 0.5	550 ± 142
A441T		1.50 ± 0.12	4.6 ± 0.5	326 ± 13
A441V		0.52 ± 0.10	1.1 ± 0.3	473 ± 216
A441F		0.34 ± 0.04	3.7 ± 0.0	92 ± 11
A441I		1.85 ± 0.06	4.8 ± 1.3	375 ± 80
				
	**NAD**^**+**^			
Wild type		0.99 ± 0.04	4.0 ± 0.1	250 ± 7
A441C		0.89 ± 0.00	2.6 ± 0.5	342 ± 76
A441S		0.88 ± 0.02	2.8 ± 0.0	314 ± 13
A441T		0.76 ± 0.02	4.0 ± 0.4	190 ± 16
A441V		0.54 ± 0.06	1.7 ± 0.2	318 ± 2
A441F		0.43 ± 0.01	5.9 ± 1.4	73 ± 17
A441I		2.11 ± 0.01	5.5 ± 0.1	382 ± 9

Initial velocities were obtained at 30°C in 50 mM HEPES-KOH buffer, pH 8.0, containing 0.1 mM EDTA. In the experiments with variable APAL, the fixed concentration of NAD^+^ was 0.2 mM, and in the experiments with variable NAD^+^ the fixed APAL concentrations were at least 10-times their app*K*_m_ values estimated for each enzyme at fixed 0.2 mM NAD^+^. The apparent kinetic parameters were estimated by non-linear regression fit of the experimental data to the Michaelis-Menten equation (saturation by NAD^+^ at fixed APAL) or to Equation 1 given in the main text (saturation by APAL at fixed NAD^+^). The values given in the Table are the mean ± standard deviation of the kinetic parameters estimated in two duplicate saturation experiments performed with enzymes from two different purification batches. Values for *k*_*cat*_ are expressed per enzyme subunit. Substrate inhibition constants for APAL are not given because they could not be accurately estimated in the concentration range used in these experiments, but the observed degree of inhibition by high APAL concentrations was roughly the same in all the enzymes.

### Structural characterization of the *So*BADH A441 mutants

**Figure 4:**
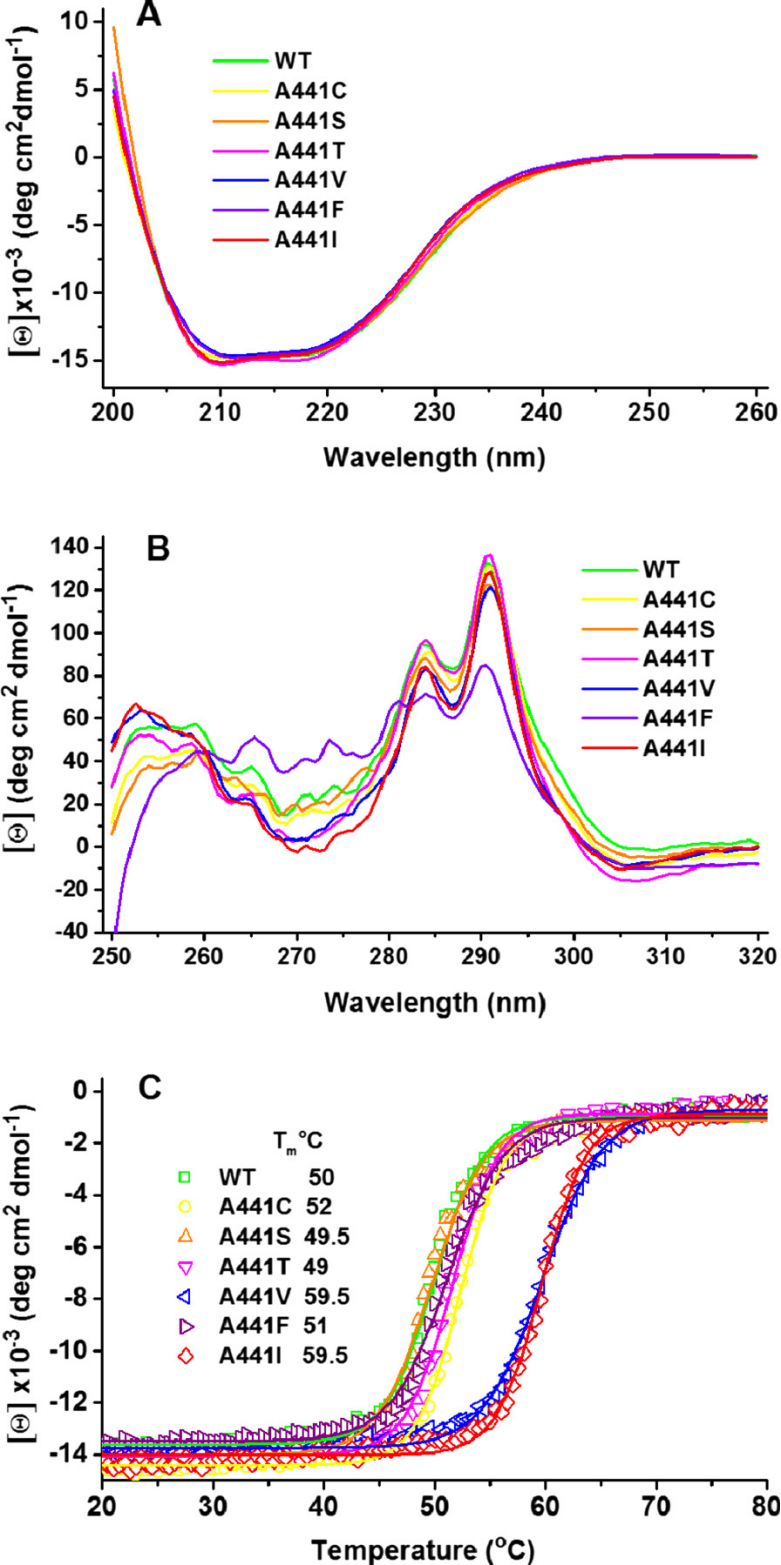
**Effects of mutation of A441 on the structural properties of *So*BADH.** Conformational characteristics of wild-type and mutant *So*BADH enzymes examined by near- **(A)** and far-UV **(B)** CD spectra. **(C)** Thermal denaturation followed by changes at 222 nm in the far-UV CD signal. The temperature range was 20–90°C and the scan rate 1.5°C/min. The solid lines represent the best fit of the thermal transition data to a sigmoidal Boltzman function by non-linear regression.

Since the two main aspects that determine the evolution of a protein are function and protein stability, we investigated whether the changes made at position 441 affect the structure and/or thermal stability of the mutant enzymes. The amino acid substitutions made at position 441 were well tolerated, and the levels of expression of soluble mutant proteins were similar to that of the wild-type enzyme (results nor shown). The mutations did not affect either the native dimeric state of the enzymes, as judged by gel filtration experiments (results not shown), or the protein secondary structure, as judged by their almost identical far-UV CD spectra (Figure 4A). Changes in the protein tertiary structure could be detected in their CD spectra in the near-UV range (Figure 4B), where the signals originate from aromatic residues. The near-UV-CD spectrum of wild-type *So*BADH shows well-defined positive maximum bands at 284 and 291 nm, characteristic of tryptophan residues, and a minimum between 260 to 280 nm [30], which were also observed in the mutant enzymes. The exception was A441F, which exhibited an altered near-UV CD spectrum with a pronounced decrease in the intensity of the peaks at 284 and 291 nm and a reduction of the deep of the trough between 260 to 280 nm. These changes probably are the result of the interaction of the Phe benzyl ring with the neighbor side chain of W456, as will be discussed below.

The stability of the mutant *So*BADH enzymes was measured by thermal denaturation, which was monitored by following the far-UV CD signal at 222 nm. As previously found with the wild-type enzyme [30], all mutant proteins were irreversibly denatured at 90°C and the melting curves exhibited monophasic transitions (Figure 4C). The irreversibility of the thermal denaturation of all enzymes precludes equilibrium thermodynamic analysis of the process. However, the use of the same measurement parameters and of the same experimental conditions allowed us to evaluate the possible effect of the changed amino acid on the mutant enzymes stability by comparing the transitions midpoints of their thermal transitions, i.e. their apparent *T*_m_ values. The estimated apparent *T*_m_ values of the A441C, A441S, A441T and A441F mutants were similar to that of the wild-type enzyme, around 50°C, but those of A441V and A441I were approximately 10°C higher (Figure 4C). These findings clearly indicate the stabilizing effect of the presence of a hydrophobic side-chain of medium or large volume inside the protein at position 441. In the case of the A441F mutant, although the side chain introduced is highly hydrophobic and the minimized model of this mutant indicates that it may make μ-stacking interactions with the side chain of W456 (see below), the strain exerted on the protein to accommodate the bulky benzyl ring of Phe, also indicated by the model, probably causes a decrease in the stability of this enzyme when compared with that of the A441I or A441V mutants. Not considering the A441F mutant, it is interesting that the differences in thermostability of the enzymes also indicate the existence of the same two groups identified by the differences in the kinetic parameters of the reaction with BAL as substrate. Clearly both effects depend on the packing of the side-chains in the region surrounding the position 441.

### Models of the *So*BADH A441 mutants

**Figure 5:**
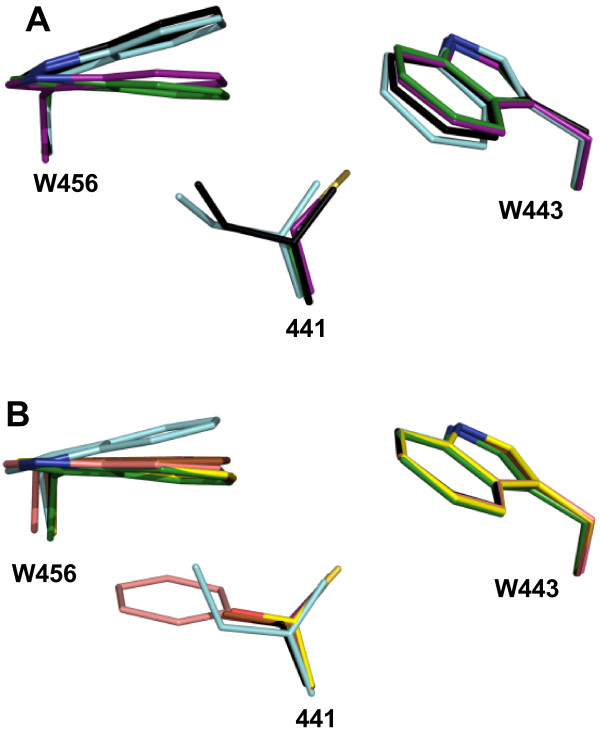
**Structural comparisons of the A441 region of ALDH10 isoenzymes. A)** Superimposition of the side-chains of residues at position 441, 443 and 456 (*So*BADH numbering) in the known crystal structures of plant ALDH10 enzymes. Side-chains are shown as sticks with oxygen atoms in red, nitrogen in blue, and sulphur in yellow. Carbon atoms are green in *So*BADH (PDB code 4A0M), cyan in *Ps*AMADH2 (PDB code 3IWJ), magenta in *Zm*AMADH1a (PDB code 4I8P), and black in *Sl*AMADH1 (PDB code 4I9B). **B)** Superimposition of the same region in the minimized models of the *in silico So*BADH mutants in which the residue at position 441 was changed. Side-chains are shown as sticks with oxygen atoms in red, nitrogen in blue, and sulphur in yellow. Carbon atoms are green in the wild-type enzyme, magenta in A441C, grey in A441S, yellow in A441T, brown in A441V, salmon in A441F, and cyan in A441I. In the figure, the wild-type and A441T models mask the A441C and A441S models, respectively. The figure was generated using PyMOL (http://www.pymol.org).

To interpret the observed kinetic and stability properties of the *So*BADH mutants, we got an estimation of the possible position and contacts in the *So*BADH structure of the changed 441 residue by performing *in silico* mutations followed by energy minimizations of the mutated structures. The results of these simulations are consistent with the known crystal structures of the ALDH10 enzymes from pea and tomato, which have Ile at position 441, and maize, which has a Cys at this position (Figure 5). This support the validity of the models of the mutant enzymes for which there is no a homolog crystal structure. When compared with the wild-type enzyme, the models of A441V and, particularly, of A441I show a similar displacement of W456 to that observed in the crystal structures of the I441-type isoenzymes of pea and tomato. This displacement causes the narrowing of the cavity where the trimethylammonium group of BAL binds, thus explaining the low BADH activity of these two mutants. On the contrary, W456 occupies almost the same position in the models of A441C, A441S and A441T than in the wild-type spinach and maize enzymes (Figure 5A and B), which is consistent with the significant BADH activity exhibited by these three mutants.

In the crystal structure of *So*BADH the side chain of A441 interacts only with the active site residue W456, but in the known structures of the other three plant ALDH10s the residue at this position also makes contacts with W443 (*So*BADH numbering) (Additional file 2: Figure S1A and Table S2). In all plant ALDH10s of known sequence, W456 is highly conserved (97.5%) and W443 strictly (100%) conserved. W433 is located at the interface between monomers and has a similar conformation in the ALDH10 crystal structures so far determined. The models of the *So*BADH A441 mutants show that the number of contacts made by the side chain of the residue at position 441 considerably increases when the size of its side chain increases (Additional file 2: Figure S1B and Table S2). Regardless of the position of the thiol group at the start of the simulation, we always obtained a model of A441C that has the thiol group pointing at W443, as in the *Zm*AMADH1a crystal structure where the sulphur of the cysteine residue at position 441 makes closer contacts with W448 (equivalent to W443 in *So*BADH) than with W461 (equivalent to W456 in *So*BADH). The serine residue in A441S can have two alternative positions: one in which the hydroxyl group is pointing at W443 and another where the hydroxyl points to W456. The first of these two possible conformations, which is similar to that adopted by Cys, is less stable since the hydroxyl in this conformation would be too far from the aromatic ring of W443 to make any interaction with it. The second conformation, which is the one of the model shown in Additional file 2: Figure S1B, is favored because the hydroxyl group makes van der Waals contacts, and possibly polar-μ interactions, with the ring of W456. In the A441T mutant, the hydroxyl group has only one possible conformation, the one that points to W456, similarly to that of the Ser in the A441S mutant model. In the A441T minimized model the distance of the hydrogen atom of the hydroxyl group from the centroid of the aromatic face of W456 ring is of 2.96 Å, suggesting the possible existence of a hydroxyl-aromatic-ring hydrogen bond of the kind described by Levitt and Perutz [31]. The Val side chain in A441V fits well in this position and makes contacts with W456. The side chain of the Ile in the mutant A441I makes almost the same contacts that those observed in the crystal structures of the pea and tomato enzymes (PDB codes 3IWJ and 4I9B, respectively), and similarly pushes the side-chain of W456. Finally, in the mutant A441F, the only possible way to accommodate the bulky benzyl group is by stacking against the aromatic ring of W456. However, the A441F model showed a clash of the F441 ring with the main chain in the region of residues P455, W456 and G457, which causes this region to move in order to accommodate the phenylalanine residue, thus narrowing the aldehyde entrance tunnel (not shown).

## Discussion

### Importance of size, polarity and conformation of the side chain at position 441 of *So*BADH for the kinetics and stability

All data in this work agree with our previous proposal that only one amino acid residue at position 441 is critical for ALDH10 enzymes to accept or reject BAL as substrate [19]. I441-type isoenzymes posses low or very low activity with BAL whereas A441- and C441-type isoenzymes exhibit high activity with BAL [19]. The *So*BADH mutants that have a residue of similar size to Ala or Cys, as A441S or A441T, exhibit a high activity with BAL, but the mutants with a bulky nonpolar residue, as A441V or A441I, have a very low activity with BAL (Figure 3). The exquisite sensitivity of *So*BADH affinity for BAL to the size of the side chain of the residue at position 441 is reflected in the finding of a *K*_m_(BAL) of the A441T mutant lower than that of the A441V. Val and Thr have similar sizes but the methyl group of Val pointing at W456 is a hydroxyl group in Thr. Since the van der Waals radius of oxygen is 0.27 Å lower than that of carbon [32], this difference would result in a lesser steric impediment of W456 to the binding of the trimethylammonium group of BAL in the A441T than in the A441V mutant. Also, the polar μ-interaction between the hydroxyl group of T441 and the aromatic ring of W456 suggested by the model would reduce the distance between these two groups, thus widening the trimethylammonium-binding cavity when compared with that of the A441V mutant. Although the van der Waals radius of oxygen is 0.39 Å lower than that of sulphur [32], the A441C mutant has a slightly but significantly lower *K*_m_(BAL) than A441S, which can be explained by the different conformation adopted by their side chains according to the models (Figure 5B and Additional file 2: Figure S1B). In the A441C mutant the atom closer to the W456 ring is a hydrogen, as is in the wild-type enzyme, whereas in the A441S mutant the position of this hydrogen is occupied by a bulkier hydroxyl group. This could explain that the A441C *So*BADH mutant has similar kinetic parameters for BAL than the wild-type enzyme. Regarding the mutant A441F, which has the bulkiest of the side chains introduced at this position, it was surprising to us that it had a lower *K*_m_(BAL) than the mutant A441I. This may be due to the stacking of the aromatic ring of F441 with that of W456, as suggested by the model, which would result in a more compact packing of both side chains, and therefore in a lesser steric impediment of W456 to the binding of the trimethylammonium group of BAL that in the A441I mutant. The movement of the main chain, also suggested by our model, and the consequent narrowing of the aldehyde-binding tunnel could explain the low *k*_cat_/*K*_m_(APAL) exhibited by this enzyme. In summary, it is not only the size but also the conformation adopted by the side chain of residue 441 what matters in determining the position of the side chain of W456, and therefore the size of the pocket where the trimethylammonium group of BAL binds and the affinity for BAL. Assuming that *K*_m_ values are an indication of affinity, the size of the residue at position 441 also appears to affect the binding of the nucleotide, although to a much lesser extent than the binding of the aldehyde, for as yet not clear reasons.

The acquisition of new functions by proteins is limited because most mutations have destabilizing effects, particularly if the mutated residue is buried [33], as is the residue at position 441. However, the findings that the variants of *So*BADH with six different residues at position 441 were correctly folded and have thermal stabilities in the range expected for mesophilic enzymes, indicate that this position is highly evolvable and able to accommodate several mutations, some of them giving rise to a new function: the BADH activity and therefore, the capacity to participate in the synthesis of the osmoprotectant GB. The higher thermal stability of the A441V and A441I mutants relative to that of the wild-type and A441C, A441S, and A441T is consistent with the extensive interactions made by the side chains of Val and Ile, as indicated by our models and the known crystal structures of ALDH10 enzymes that contain Ile at this position, and with the hydrophobicity of these two residues. In the case of the A441F mutant the strain exerted on the protein by the bulky benzyl ring of Phe probably causes a decrease in the stability of this enzyme when compared with that of the A441I or A441V mutants.

### Evolution of plant ALDH10 enzymes

One of the strategies used by plants to respond to the demands of their environment involves the evolution of novel metabolic pathways by recruiting enzymes from old ones. The phylogenetic evidence presented here indicates that this is the case of the enzymes exhibiting BADH activity that participate in the synthesis of the osmoprotectant glycine betaine (GB), which in angiosperms evolved from I441-type ALDH10 enzymes.

Our results show that non-flowering plants possess only one *ALDH10* gene expressing an I441-type enzyme, but flowering plants (monocots plus eudicots) have a variable number of *ALDH10* genes coding for I441-, A441- or C441-type isoenzymes. The presence of additional gene copies in monocots and eudicots can be explained by several small- and large-scale duplications, which were accompanied by gene losses and genome rearrangements [34]. Indeed, the distribution of the duplicated ALDH10 enzymes that can be seen in the phylogenetic tree in Figure 1 is in agreement with previous observations: (i) various eudicot families independently underwent genome duplication [35,36]; (ii) one whole-genome duplication occurred early in the monocot lineage after its divergence from the eudicot lineage [37]; and (iii) recent duplication events took place in maize, soybean and grape [34]. Duplication is a prominent feature of the plant genomic architecture, and much of plant diversity may have arisen following the duplication and adaptive specialization of pre-existing genes [38]. In this context, duplication of the *ALDH10* gene in some plants allowed a functional specialization when I441 mutated to A441 or C441 in one of the two copies of the duplicated gene, as proposed by Díaz-Sánchez *et al.*[19]. The ancient enzymes most probably had the same AMADH activity that the present-time I441-type isoenzymes, but they may have had also a vestigial BADH activity, which was improved during the evolution of plants via an evolutionary pathway of sequential single mutations of the duplicates. In this way, the A441- or C441-type isoenzymes acquired an extra BADH activity, necessary for GB synthesis, while the original peroxisomal I441-type isoenzymes remained as AMADHs devoid of BADH activity and most likely retained their metabolic role. Although the gain of the BADH activity does not imply the loss of the other AMADH activities when assayed *in vitro* ([19]; this work), the above observations indicate that the physiological functions of these isoenzymes are not interchangeable. Indeed, in many plant species the duplication of the *ALDH10* gene did not result in functional redundancy, since the duplicated genes not only derived in enzymes with BADH activity but also in non-peroxisomal I441-type AMADH isoenzymes, as in the Solanaceae, Malvaceae, Rosacease and Brassicaceae families, whose different cellular location probably allows them to perform a different physiological function than the peroxisomal ones. This is one possible reason for the permanence of the duplicate genes that encode non-peroxisomal I441-type isoenzymes.

Our phylogenetic analysis also shows that all known plant genomes contain at least one *ADH10* gene coding for a peroxisomal I441-type isoenzyme, which is the one present even in those plants with a sequenced genome that possess only one *ALDH10* gene. This underscores the importance of the biochemical processes in which these peroxisomal I441-type isoenzymes participate, and indicate that, as mentioned above, *in vivo* they cannot be replaced by the ALDH10 isoenzymes that have gained the extra BADH activity, i.e. the A441- or C441-type isoenzymes. Together, our findings strongly support that the activity of the peroxisomal I441-type isoenzymes is essential for the plant, which is not unexpected considering that the AMADH activity of these enzymes is involved in the catabolism of polyamines taking place in the peroxisome [39]. As mentioned above, the cytosolic I441-type isoenzyme may be involved in other physiological processes, for instance the synthesis of several osmoprotectants, such as 4-aminobutyrate, β-alanine, or carnitine by oxidizing ABAL, APAL or TMABAL. The different intracellular location of the ALDH10 isoenzymes, predicted and in some cases experimentally proven, suggest that there are two kinds of the I441-type—peroxisomal (the commonest) and cytosolic—, both devoid of significant BADH activity, and possibly two kinds of the A441- and C441-type—chloroplastic and cytosolic in the first case, and peroxisomal and cytosolic in the second—, all of them having BADH activity.

Regarding the intracellular location, the Amaranthaceae A441-type isoenzymes most likely are chloroplastic, as those from spinach [40] and sugarbeet [28], although they lack a typical chloroplast-targeting signal [14]. Therefore, in the Amaranthaceae the synthesis of GB most likely takes place in the chloroplasts, as experimentally proven for spinach [13]. This is in accordance with the chloroplastic location of the CMO enzyme in this plant [41] and with the fact that the CMO activity requires the electrons provided by reduced ferredoxin [17] and plant ferredoxins are plastidic proteins [42]. But in the Poaceae plants that have the C441-type isoenzyme, GB synthesis may take place in the cytosol—since some of these isoenzymes are non-peroxisomal and do not have a clear chloroplast-leading signal—or in peroxisomes, since other C441-type isoenzymes have the peroxisomal signal. Indeed, it has been suggested that in barley the synthesis of GB may take place in the peroxisome, given the finding of a peroxisomal CMO in this plant [43]. But this proposal is at odds with the proven non-peroxisomal location of the C441-type ALDH10 isoenzyme of barley [9], and with the fact that peroxisomal plant ferredoxins have not been so far reported. Regardless of the peroxisomal or non-peroxisomal CMO location, it seems probable that the need of transport into the peroxisomes of either choline or BAL limits the synthesis of GB in those plants with C441-type peroxisomal isoenzymes in comparison with those in which this isoenzyme is non-peroxisomal. This is in full agreement with the finding that the level of GB accumulation observed in cereal plants that have a predicted peroxisomal C441-type isoenzyme—maize, sorghum and foxtail millet—is much lower than that in plants that have the non-peroxisomal C441-type, as wheat and barley [44-46]. It could be predicted that other Poaceae species which have a moderate ability to accumulate GB, such as *Pennisetum*[44] and *Panicum*[46], have peroxisomal C441-type isoenzymes, while those that are high GB accumulators, such as *Secale*[44,46], have the non-peroxisomal C441-type isoenzyme.

Since the ability to synthesize the osmoprotectant GB protects the plant against the most frequent environmental stresses such as salinity, drought, and low temperatures, as well as indirectly against other stresses that usually accompany the formers, such as oxidative stress and high temperatures (rev. in [47,48]), it is clear that the presence of A441- or C441-type isoenzymes provides a strong adaptive advantage. And not only to halophytes, which grow in habitats where saline soils are prevalent, but also to mesophytes, which may experience sporadic episodes of water deficit or freezing temperatures. This, together with the relatively easiness of the evolutionary process, explains why the A441- and C441-type isoenzymes evolved independently several times through the evolution of flowering plants. However, the A441- or C441-type isoenzymes exhibit a limited phyletic distribution (Figures 1B and 1C), which agrees with the finding that GB accumulation is also restricted to some eudicot and monocot families [19]. This finding further support the proposal that a significant BADH activity is essential for GB accumulation [19]. Indeed, a significant BADH activity would be necessary not only to produce the levels of GB needed for an osmoprotectant, but also to prevent the accumulation of the BAL—which is produced by CMO—up to toxic concentrations. But although the BADH activity is a *sine qua non* condition for the plant to become a GB-accumulator, the current experimental evidence, obtained studying wild-type GB-accumulator plants and transgenic non-accumulator plants that express *BADH* or *CMO* genes (rev in [1]), suggests that the acquisition of the ability to synthesize GB by evolving a BADH activity in some plant ALDH10 enzymes should have been accompanied by other adaptations, such as: (i) the gain of a significant CMO activity; (ii) the adequate intracellular targeting of both the CMO and BADH enzymes; (iii) the regulation by osmotic stress signals of the expression of the *CMO* and *BADH* genes, as well as of those coding for critical enzymes of the choline biosynthetic pathway; and (iv) probably also, the development of an efficient choline and GB transport through intracellular membranes.

### Evolutionary pathway for the acquisition of BADH activity by plant ALDH10 enzymes

Our results indicate that plant ALDH10 enzymes may have followed several evolutionary pathways for the acquisition of BADH activity, particularly the ones involving any of the parsimonious intermediates with Val, Thr, Ser at position equivalent to 441 of *So*BADH. The evolutionary intermediate carrying F441 would have a diminished AMADH activity, as judged by the low *k*_cat_/*K*_m_ values of the *So*BADH A441F mutant, which could make the route though it less probable. But as the enzymes that undergo these mutations were coded by gene duplicates and the original I441-type enzymes were conserved, the plant would not have experienced any deleterious effect even if the mutation to Phe took place. But this mutation would have not provided any advantage to the plant either, in contrast to the Ala o Ser mutations, and even to the mutation that led to non-peroxisomal I441-type isoenzymes. Thus, the Phe441 mutation could have been eliminated during evolution. Interestingly, natural ALDH10s of the T441-type (2 sequences) and V441-type (3 sequences), which the intermediary mutants in the parsimonious pathway to A441 from I441, were found within the plant ALDH10 protein sequences retrieved from public databases. We anticipate that enzymes of the S441-type will be found when more monocot sequences are known. The relatively high frequency of A441-type ALDH10 isoenzymes may result from a bias in the available reports, since most of the studies have been carried out in halophytic plants in which these enzymes participate in their osmotolerance mechanisms. Regarding the C441-type isoenzymes, they are present in cereal plants, which have been extensively studied for their high agronomical interest. Indeed, in cereals an evolutionary process directed by humans to increase their tolerance to drought or saline stress may have occurred.

The mutation of the original Ile residue at this position to Val, Thr or Ser could be considered as neutral since it would not have had any deleterious effect on the primitive AMADH function, as judged by the *k*_cat_/*K*_m_(APAL) values of the mutant enzymes studied here. But while in the case of the mutation to Val the resulting enzyme would still be an AMADH devoid of significant BADH activity, in the case of the mutation to Thr or Ser the enzyme would have experienced an important increase in the vestigial BADH activity of the original enzyme, which would make these enzymes true BADHs. The kinetic data of the mutant enzymes suggest that the BADH activity of the T441-type or the S441-type intermediates would be increased if a second mutation to Ala or Cys took place. The same BADH activity would be gained after the mutation of Val to Ala. These increases could be of enough physiological importance to explain the selection of the enzymes with this second mutation to either Ala or Cys, which in turn would explain, at least in part, the relatively higher frequency of the A441- or C441-type isoenzymes over that of the V441-, T441-, or S441-type.

**Figure 6:**
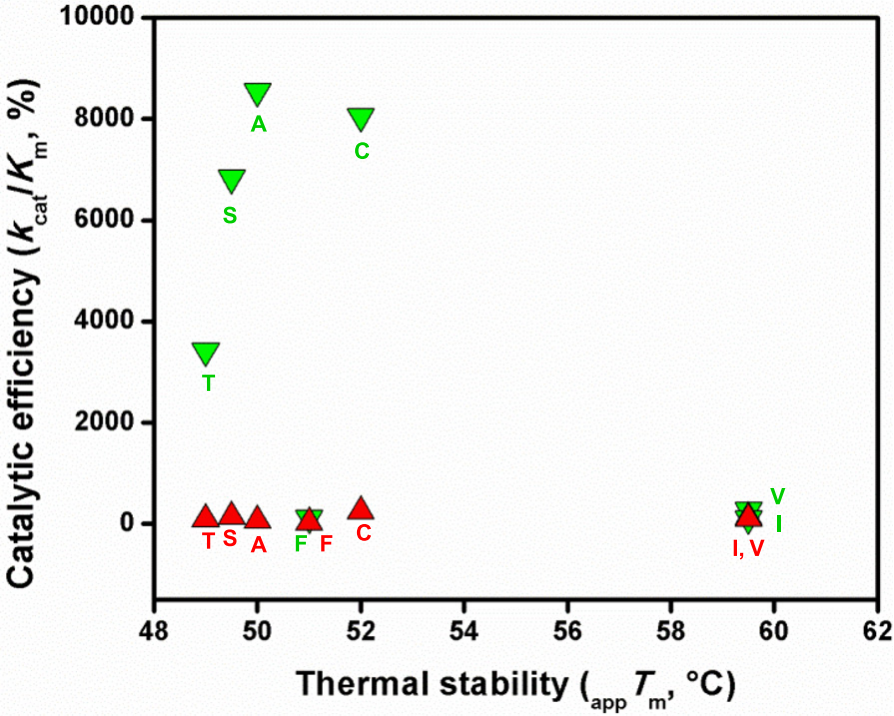
**Comparison of thermal stabilities and catalytic efficiencies of wild-type and A441 *So*BADH mutants.** The percentage of catalytic efficiency for the oxidation of BAL (green down triangles) or APAL (red up triangles) was calculated from the *k*_cat_/*K*_m_ values given in Table 1 and Table 2, respectively, taking as 100% the values of the A441I mutant, which was assumed to represent the ancestral AMADH enzyme. Thermal stability data (apparent *T*_m_ values) are those given in Figure 4. *So*BADH Enzymes: A, wild-type; C, A441C; S, A441S; T, A441T; V, A441V; F, A441F; I, A441I.

The decrease in the thermal stability of those ALDH10 enzymes that underwent the mutational event by which they acquire BADH activity—as suggested by our results with the *So*BADH mutants at position 441—would not compromise their stability under physiological conditions. Neither would be the folding affected, as judged by the similar level of expression of these mutant enzymes as soluble proteins in *E. coli*. As a summary, Figure 6 shows the comparison of the thermal stabilities and catalytic efficiencies of the seven *So*BADH proteins studied in this work—the wild type and six mutants— to illustrate the possible fitness landscape of the evolution of the BADH activity within the ALDH10 family. As can be seen, the change of the Ile at position 441 does not affect the catalytic efficiency with APAL as substrate but it has a profound effect on the catalytic efficiency with BAL. The thermostability cost of this change would be surpassed by the advantage of the gain of the BADH activity. Indeed, this may be the reason for the scarcity of V441-type isoenzymes in the present-time plant ALDH10s, which is rather surprising given that the change of I441 for V441 has almost no effect either on AMADH activity or protein stability. It can be speculated that a subsequent mutation to A441 is so advantageous for the plant that most of the V441 enzymes evolved to this final stage.

## Conclusions

The phylogenetic, biochemical, and structural evidence presented here support relatively smooth evolutionary processes for the gain of the BADH activity in plants, and therefore for the acquisition of the potential to synthesize the osmoprotectant GB, since evolution took place in duplicated genes and the four parsimonious evolutionary intermediates are functionally and structurally feasible, particularly three of them. Our results strongly support that ancestral genes encoding peroxisomal I441-type isoenzymes devoid of BADH activity mutated in several occasions during terrestrial plant evolution, so that the present time A441- or C441-type isoenzymes that exhibit BADH activity do not form a monophyletic group inside the ALDH10 protein family. We also provide additional evidence of the critical role played by the residue at position 441 (*So*BADH numbering) in the affinity for BAL of plant ALDH10 enzymes. Finally, our findings about the differences in intracellular location of the isoenzymes with BADH activity indicate that the peroxisomal synthesis of GB is constrained by some factor(s), and explain the known fact that there are high and medium-GB accumulator plants, the former having non-peroxisomal A441- or C441-type isoenzymes, the latter being those with a peroxisomal C441-type.

## Methods

### Chemicals and biochemicals

Betaine aldehyde chloride, the diethylacetal of APAL and NAD^+^ were obtained from Sigma-Aldrich Química, S.A. de C.V. (Toluca, México). APAL was freshly prepared by hydrolyzing its diethylacetal form as described by Flores and Filner [49]. The exact concentration of the resulting free APAL was determined in each experiment by the amount of NADH produced after its complete oxidation in the reaction catalyzed by *So*BADH in the presence of an excess of NAD^+^.

### Site-directed mutagenesis, production and purification of wild-type and mutant *So*BADH enzymes

The plasmid pET28-*So*BADH, containing the full sequence of the spinach *BADH* gene and a N-terminal His-tag [19], was used as template for site-directed mutagenesis, which was performed via polymerase chain reaction (PCR) using the Quick Change XL-II Site Directed Mutagenesis system (Agilent) and the following mutagenic primers: A441V, GAAGGCTCTAGAAGTTGGA*GTT*GTTTGGGTTAATTGCTCAC (forward) and TTGTGAGCAATTAACCCAAAC*AAC*TCCAACTTCTAGAGCC (reverse); A441S, GAAGGCTCTAGAAGTTGGA*ACT*GTTTGGGTTAATTGCTCAC (forward) and TTGTGAGCAATTAACCCAAAC*AGT*TCCAACTTCTAGAGCC (reverse); A441T, GAAGGCTCTAGAAGTTGGA*ACC*GTTTGGGTTAATTGCTCAC (forward) and TTGTGAGCAATTAACCCAAAC*GGT*TCCAACTTCTAGAGCC (reverse); A441F, GAAGGCTCTAGAAGTTGGA*TTT*GTTTGGGTTAATTGCTCAC (forward) and TTGTGAGCAATTAACCCAAAC*AAA*TCCAACTTCTAGAGCC (reverse);. The non-complementary mutagenic codons are in italics. Mutagenesis was confirmed by DNA sequencing. The expression and purification of the recombinant proteins were carried out as reported [19]. Protein concentrations were determined spectrophotometrically, using the molar absorptivity at 280 nm of 86,400 M^-1^ cm^-1^ deduced from the amino acid sequence by the method of Gill and von Hippel [50].

### Activity assay and kinetic characterization of the wild-type and mutant *So*BADH enzymes

Steady-state initial velocities of wild-type *So*BADH and its mutants were measured spectrophotometrically as reported [19] at pH 8.0 using 0.2 mM NAD^+^ and variable concentrations of BAL or APAL. The fixed concentration of NAD^+^ used in these experiments was saturating, as indicated by the finding of very close *k*_cat_ values in experiments where the nucleotide was the variable substrate and the aldehyde kept constant at a concentration ten times its *K*_m_ value. All assays were initiated by addition of the enzyme. Each saturation curve was determined at least in duplicate using enzymes from two different purification batches. Initial velocity data were analyzed by non-linear regression calculations using the Michaelis-Menten equation. Equation 1 was used for the APAL saturation data, which exhibited substrate inhibition:

(1) $$v/\left[E\right]=k_{\mathrm{cat}}\left[S\right]/\left\{K_{m}+\left[S\right]\left(1+\left[S\right]/K_{\mathrm{IS}}\right)\right\}$$

where *v* is the experimentally determined initial velocity, [E] is the enzyme concentration, *k*_*cat*_ is the first order rate constant for product formation at saturating substrate (equal to the maximal velocity, *V*_max_, divided by the enzyme concentration), [S] is the concentration of the variable substrate, *K*_m_ is the concentration of substrate at half-maximal velocity, and *K*_IS_ is the substrate inhibition constant. Although substrate inhibition of *So*BADH by aminoaldehydes is partial [19], the highest substrate concentration used in our experiments did not allow distinguishing between total and partial inhibition, and therefore Equation 1 for total inhibition was used.

### Circular dichroism spectroscopy

CD signals were recorded with a Jasco J-715 spectropolarimeter (Jasco Inc., Easton, MD) equipped with a Peltier-type temperature control system (Model PTC-423S) and calibrated with *d*-10-(+)-camphorsulfonic acid. Near-UV (250–320 nm) and far-UV (200–250 nm) CD spectra were recorded for samples of 0.25 or 1.0 mg/mL protein concentration, respectively, placed in quartz cuvettes of 1.0-cm and 0.1-cm path length, respectively. Data were collected at 0.5 nm (near-UV) or 1.0 nm (far-UV) intervals, a bandwidth of 1.0 nm and at a scan rate of 20 nm/min. Spectra were averaged over 5 scans and the average spectrum of a reference sample without protein was subtracted. The observed ellipticities were converted to mean residue ellipticities [Θ] on the basis of a mean molecular mass per residue of 109.1. Thermal-induced protein denaturation was monitored by following the changes in ellipticity at 222 nm by increasing the temperature from 20 to 90°C at a constant rate of 1.5°C/min. Determination of apparent *T*_m_ values was performed by non-linear regression fit of the data to a single Bolztman sigmoidal function. ORIGIN software (OriginLab Corp.) was used for data analysis and display.

### *In silico* mutagenesis and modeling

Mutations in the crystal structure of the *So*BADH (PDB code 4A0M) at position 441 were generated *in silico* using the standard rotamer library of Coot [51]. Mutant models were subjected to a 1000-step energy-minimization process using the Amber force field parameters in the UCSF Chimera [52].

### Sequence analyses

ALDH10 amino acid and nucleotide sequences were retrieved by Blast searches at the NCBI site [53] (http://blast.ncbi.nlm.nih.gov/Blast.cgi) or Phytozome v9.1 database ([54]; http://www.phytozome.net/). Progressive multiple amino acid sequence alignments were performed with ClustalX version 2 ([55], http://www.clustal.org/clustal2/) using as a guide a structural alignment constructed with the VAST algorithm [56] that included all non-redundantALDH10 protein structures in the PDB [57]. Amino acid sequence alignments were corrected manually using BioEdit ([58], http://www.mbio.ncsu.edu/bioedit/bioedit.html) according to gapped BLASTP results. To identify protein sequences as members of the ALDH10 family the phylogenetic analyses previously performed by Julián-Sánchez *et al*. [59] were used as a reference. Phylogenetic analyses were conducted using the MEGA5 software ([60]; http://www.megasoftware.net). Four methods were used to infer phylogenetic relationships: maximum likelihood (ML), maximum parsimony (MP), minimum evolution (ME), and neighbor joining (NJ). The amino acids substitution model described by Whelan and Goldman [61] using a discrete Gamma distribution with five categories, was chosen as the best substitution model, since it gave the lowest Bayesian Information Criterion values and corrected Akaike Information Criterion values [62] in MEGA5 [60]. The gamma shape parameter value (+G parameter = 1.1824) was estimated directly from the data with MEGA5. Confidence for the internal branches of the phylogenetic tree, obtained using ML method, was determined through bootstrap analysis (500 replicates each).

## Abbreviations

ALDH: Aldehyde dehydrogenase; ABAL: 4-Aminobutyraldehyde; AMADH: Aminoaldehyde dehydrogenase; APAL: 3-Aminopropionaldehyde; BADH: Betaine aldehyde dehydrogenase; BAL: Betaine aldehyde; CMO: Choline monooxygenase; GB: Glycine betaine; PDB: Protein data bank; *Ps*AMADH: Pea AMADH; *Sl*AMADH: Tomato AMADH; *So*BADH: Spinach BADH; TMABAL: 4-Trimethylaminobutyraldehyde; WT: Wild type; *Zm*AMADH: Maize AMADH.

## Competing interests

All the authors declare that they have no competing interests.

## Authors’ contributions

RAMC conceived, designed and coordinated the study, analyzed kinetic and structural data, and wrote the manuscript. HRR and AJS carried out the phylogenetic analysis, interpreted and wrote the results of this analysis, and contributed to the general discussion and writing of the manuscript. CMJ constructed and purified the mutant proteins and performed the kinetic experiments. GGR carried out the CD and thermostability experiments and analyzed these data. LGS made the *in silico* mutants, constructed their models and helped with the making of the figures. All authors read and approved the final manuscript.

## Supplementary Material

Additional file 1: Table S1 Aldehyde dehydrogenases identified as members of the ALDH10 family. Click here for file

Additional file 2: Figure S1 Interactions of the residue at position equivalent to A441 of *So*BADH. **Table S2.** Distances of the side-chain atoms of the residue at position 441 to their closest neighbors. Click here for file
