# Supplementary material for: Exploring the evolutionary route of the acquisition of betaine aldehyde dehydrogenase activity by plant ALDH10 enzymes: implications for the synthesis of the osmoprotectant glycine betaine
Source: BMC Plant Biol. 2014 May 29;14:149. doi: 10.1186/1471-2229-14-149 (PMC4046141; doi:10.1186/1471-2229-14-149)
Supplement: Additional file 1: Table S1 — Aldehyde dehydrogenases identified as members of the ALDH10 family. [file 1471-2229-14-149-S1.pdf]

Table S1. Aldehyde dehydrogenases identified as members of ALDH10 family
Organisms with a complete sequenced genome are shaded in light blue. Phytozome data are colored green and NCBI data black.

**Organism**<br>**Lineage**<br>**Genome assembly**<br>**Number of genes**	**ALDH10**<br>**Protein accession number**<br>**Gene locus**<br>**Amino acid sequence length**
**Plants**	
**Chlorophyta**	
**Mamiellophyceae**	
Micromonas pusilla CCMP1545<br>Chlorophyta; Mamiellophyceae; Mamiellales<br>Phytozome: partially finished v3 genome assembly and annotation<br>10 660 total loci containing protein-coding transcripts	XP_003064309<br>(C1N8N9)<br>MicpuC2.e_gw1.17.47.1<br>(MICPUCDRAFT_23534)<br>505 aa
Micromonas pusilla RCC299<br>Chlorophyta; Mamiellophyceae; Mamiellales<br>Phytozome: finished v3 genome assembly and annotation<br>17 chromosomes<br>10 103 total loci containing protein-coding transcripts	Absent
Ostreococcus lucimarinus CCE9901<br>Chlorophyta; Mamiellophyceae; Mamiellales<br>Phytozome: finished v.2 genome assembly and annotation<br>21 chromosomes<br>7,796 total loci containing protein-coding transcripts	XP_001420154<br>estExt_Genewise_ext.C_Chr_100439<br>OSTLU_50750<br>Chromosome 10<br>523 aa
Ostreococcus tauri<br>Chlorophyta; Mamiellophyceae; Mamiellales<br>8 110 genes	CAL56178<br>XP_003081654<br>Ot10g02210<br>Chromosome 10<br>506 aa
**Trebouxiophyceae**	
Coccomyxa subellipsoidea C-169<br>Chlorophyta; Trebouxiophyceae; Coccomyxaceae<br>Phytozome: improved v2 genome assembly and annotation<br>9 629 total loci containing protein-coding transcripts	EIE20955<br>I0YRD6<br>fgenesh1_pm.14_#_152<br>COCSUDRAFT_37718<br>498 aa
**Chlorophyceae**	
Chlamydomonas reinhardtii (strain: CC-503 cw92 mt+)<br>(Chlamydomonas smithii)<br>Chlorophyta; Chlorophyceae; Chlamydomonadales;<br>Chlamydomonadaceae<br>Phytozome: v5.3.1 of Chlamydomonas annotations<br>17 linkage groups (chromosomes)<br>12,264 stable gene (13,450 stable transcript)	XP_001699134<br>A8JAX9<br>Cre13.g605650<br>CHLREDRAFT_24394<br>504 aa
Volvox carteri f. nagariensis (strain: Eve, forma: nagariensis)<br>(Green alga)<br>Chlorophyta; Chlorophyceae; Chlamydomonadales; Volvocaceae<br>Phytozome: version 2, 8x genome assembly and annotation<br>14,971 total loci containing protein-coding transcripts<br>(314 total alternatively spliced transcripts)	XP_002947147<br>D8TLH8<br>Vocar20010574m.g<br>VOLCADRAFT_73155<br>503 aa
**Streptophyta**	

Organism<br>Lineage<br>Genome assembly<br>Number of genes	ALDH10<br>Protein accession number<br>Gene locus<br>Amino acid sequence length
**Embriophyta**	
Physcomitrella patens subsp. patens (Moss)<br>Streptophyta; Embryophyta; Bryophyta; Bryophytina; Bryopsida; Funariidae; Funariales; Funariaceae<br>Phytozome: v3.0 assembly (preliminary)<br>27 chromosomes<br>26610 loci containing protein-coding transcripts	XP_001756623<br>A9RQZ4<br>PHYPADRAFT_204867<br>507 aa
Selaginella moellendorffii (Spikemoss)<br>Streptophyta; Embryophyta; Tracheophyta; Lycopodiophyta; Isoetopsida; Selaginellales; Selaginellaceae<br>Phytozome: v1.0 Dec 20, 2007 FilteredModels3 annotation<br>27 chromosomes<br>22 285 protein-coding transcripts	XP_002974519<br>D8RTU8<br>174224<br>SELMODRAFT_174224<br>503 aa<br><br>XP_002990779<br>D8T5B3<br>SELMODRAFT_272160<br>503 aa<br>(redundant copy because the assembled genome includes two haplotypes)
**Gymnosperm**	
Picea sitchensis (Sitka spruce) (Pinus sitchensis)<br>Tracheophyta; Spermatophyta; Coniferopsida; Coniferales; Pinaceae	ABK24463<br>A9NV02<br>(ABK24261)<br>503aa
**Angiosperm**	
**Liliopsida (monocots)**	
Aegilops tauschii (Tausch's goatgrass)<br>Tracheophyta; Spermatophyta; Magnoliophyta; Liliopsida; Poales; Poaceae; BEP clade; Pooideae; Triticeae	EMT10403<br>M8C5I2<br>F775_31015<br>503 aa
Aeluropus lagopoides<br>Liliopsida; Poales; Poaceae; PACMAD clade; Chloridoideae; Cynodonteae; Aeluropinae	AEV53927<br>G9JNB0<br>334 aa (fragment)
Agropyron cristatum<br>(Crested wheatgrass) (Bromus cristatus)<br>Liliopsida; Poales; Poaceae; BEP clade; Pooideae; Triticeae	ACZ67850<br>D2K6H6<br>394 aa (fragment)
Brachypodium distachyon<br>Liliopsida; Poales; Poaceae; BEP clade; Pooideae; Brachypodieae<br>BioProject PRNJ74771<br>v1.0 8x assembly<br>5 chromosomes<br>26,552 loci containing protein-coding transcripts	XP_003579919<br>LOC100826926<br>Chromosome 5<br>506 aa<br><br>XP_003574495<br>LOC100845613<br>Chromosome 3<br>501 aa

**Organism**<br>**Lineage**<br>**Genome assembly**<br>**Number of genes**	**ALDH10**<br>**Protein accession number**<br>**Gene locus**<br>**Amino acid sequence length**
Hordeum brevisubulatum<br>Liliopsida; Poales; Poaceae; BEP clade; Pooideae; Triticeae	AAS66641<br>505aa
Hordeum vulgare subsp. vulgare (domesticated barley)<br>Tracheophyta; Spermatophyta; Magnoliophyta; Liliopsida; Poales; Poaceae; BEP clade; Pooideae; Triticeae /cultivar="Haruna-nijyo	BAB62846<br>BBD2<br>503 aa<br><br>BAB62847<br>BBD1<br>506 aa
Hordeum vulgare<br>Liliopsida; Poales; Poaceae; BEP clade; Pooideae; Triticeae /cultivar: 116 Jeonju Native Korca	Q40024<br>LOC548296<br>505 aa<br>(BAB62847 ortolog)
Leymus chinensis<br>Liliopsida; Poales; Poaceae; BEP clade; Pooideae; Triticeae	BAD86758<br>LcBADH2<br>502 aa<br><br>BAM10432<br>BAD86757<br>LcBADH1<br>506 aa
Lolium perenne (Perennial ryegrass)<br>Liliopsida; Poales; Poaceae; BEP clade; Pooideae; Poeae; Loliinae	AFA36547<br>288aa (fragment)
Ophiopogon japonicus<br>Liliopsida; Asparagales; Asparagaceae; Nolinoideae	ABG34273<br>Q153G6<br>BADH<br>500 aa
Oryza sativa Japonica Group<br>Tracheophyta; Spermatophyta; Magnoliophyta; Liliopsida; Poales; Poaceae; BEP clade; Ehrhartoideae; Oryzeae<br>BioProject PRJNA13141:<br>30534 genes (12 chromosomes)<br>28555 proteins<br>BioProject PRJNA13139<br>37032 genes (12 chromosomes)<br>35394 proteins	XP_482470<br>NP_001061833<br>BADH2<br>Os08g0424500<br>(Phytozome: LOC_Os08g32870)<br>Chromosome 8<br>503 aa<br><br>O24174<br>NP_001053016<br>Os04g0464200<br>BADH1<br>(Phytozome: LOC_Os04g39020)<br>Chromosome 4<br>505 aa

Organism<br>Lineage<br>Genome assembly<br>Number of genes	ALDH10<br>Protein accession number<br>Gene locus<br>Amino acid sequence length
Oryza sativa Indica Group (long-grained rice)<br>Tracheophyta;Spermatophyta; Magnoliophyta; Liliopsida; Poales; Poaceae; BEP clade; Ehrhartoideae; Oryzeae<br>BioProject PRJN361<br>39285 genes (12 chromosomes)<br>37358 proteins	CM000133<br>Chromosome 8<br>503 aa<br>(unreported gen)<br><br>EEC77423<br>B8AV58<br>OsI_16212<br>Chromosome 4<br>505 aa<br>Almost identical to:<br>ABB83473<br>505 aa
Pandanus amaryllifolius<br>Liliopsida; Pandanales; Pandanaceae	AFD62259<br>H9BS79<br>BADH2<br>387 aa (fragment)
Setaria italica (foxtail millet)<br>Liliopsida; Poales; Poaceae; PACMAD clade; Panicoideae; Paniceae<br>chromosome-scale release of the 8.3x whole genome shotgun assembly (98.9% of the sequence data is represented in the 9 pseudomolecules).<br>35,471 loci containing protein-coding transcripts	Si009902m<br>Si009902m.g<br>505 aa<br><br>Si013592m<br>Si013592m.g<br>505 aa
Sorghum bicolor (sorghum)<br>Liliopsida; Poales; Poaceae; PACMAD clade; Panicoideae; Andropogoneae<br>NCBI: BioProject PRJNA38691, PRJNA13876; Assembly: Sorbi1<br>10 chromosomes<br>33,080 genes<br>Phytozome: v1.0 release, comprising the Sbi1 assembly and Sbi1.4 gene set<br>20 chromosomes<br>34,496 loci containing protein-coding transcripts	AAC49268_NC012875<br>SORBIDRAFT_06q019200<br>Chromosome 6<br>506 aa<br><br>XP_002444357<br>SORBIDRAFT_07g020650<br>Chromosome 7<br>505 aa
Triticum aestivum (bread wheat)<br>Liliopsida; Poales; Poaceae; BEP clade; Pooideae; Triticeae	AAL05264<br>CBK51339<br>503 aa
Triticum urartu (Red wild einkorn)<br>Tracheophyta; Spermatophyta; Magnoliophyta; Liliopsida; Poales; Poaceae; BEP clade; Pooideae; Triticeae	EMS68665<br>M8ATW1<br>TRIUR3_04819<br>417 aa<br><br>EMS48376<br>M7YN64<br>TRIUR3_05640<br>544 aa

**Organism**<br>**Lineage**<br>**Genome assembly**<br>**Number of genes**	**ALDH10**<br>**Protein accession number**<br>**Gene locus**<br>**Amino acid sequence length**
Zea mays<br>Liliopsida; Poales; Poaceae; PACMAD clade; Panicoideae; Andropogoneae<br>BioProject B73RefGen_V2<br>10 chromosomes<br>39 454 genes	AAT70230<br>NP_001105781<br>LOC606443<br>AMADH1B<br>505 aa<br><br>NP_001157807<br>(PDB: 4I8P)<br>C0P9J6<br>gpm154<br>AMADH1A<br>LOC100302679<br>505 aa<br><br>NP_001157804<br>AMADH2<br>506 aa
Zoysia tenuifolia<br>Liliopsida; Poales; Poaceae; PACMAD clade; Chloridoideae; Zoysieae; Zoysiinae	BAD34955<br>clone="18<br>BAD34954<br>BAD34947<br>504 aa<br><br>BAD34957<br>clone="ZBD1"<br>BAD34952<br>BAD34953<br>507 aa
**Eudicotyledons**	
Amaranthus hypochondriacus (grain amaranth)<br>eudicotyledons; core eudicotyledons; Caryophyllales; Amaranthaceae	O04895<br>AAB58165<br>BADH4<br>Chloroplastic<br>501 aa<br><br>AAB70010<br>O22467<br>BADH17<br>500 aa
Ammopiptanthus mongolicus<br>eudicotyledons; core eudicotyledons; rosids; fabids; Fabales; Fabaceae; Papilionoideae; Thermopsideae	ABC86862<br>Q2I693<br>241aa (fragment)

Organism<br>Lineage<br>Genome assembly<br>Number of genes	ALDH10<br>Protein accession number<br>Gene locus<br>Amino acid sequence length
Aquilegia coerulea Goldsmith (Rocky mountain columbine; Colorado blue columbine)<br>Streptophyta; Embryophyta; Tracheophyta; Spermatophyta; Magnoliophyta; eudicotyledons; Ranunculales; Ranunculaceae<br>Phytozome: initial 8X unmapped genome assembly and the version 1.1 annotation<br>24,823 loci containing protein-coding transcripts	Aquca_002_00337<br>503 aa
Arabidopsis lyrata subsp. lyrata (Lyre-leaved rock-cress)<br>eudicotyledons; core eudicotyledons; rosids; malvids; Brassicales; Brassicaceae<br><br>Phytozome: includes JGI release v1.0<br>32670 protein-coding transcripts	XP_002877599<br>D7LRJ5<br>ARALYDRAFT_906054<br>937567<br>ALDH10A9<br>503 aa<br><br>XP_002889000<br>D7KSI7<br>ARALYDRAFT_895359<br>926872<br>ALDH10A8<br>501 aa
Arabidopsis thaliana (thale cress)<br>eudicotyledons; core eudicotyledons; rosids; malvids; Brassicales; Brassicaceae; Camelineae<br><br>Phytozome; includes TAIR annotation release 10 of the Arabidopsis thaliana genome release 9<br>27416 loci containing protein-coding transcripts	AAL34161<br>NP_190400<br>ALDH10A9<br>AT3G48170<br>Chromosome 3<br>Peroxisome<br>503 aa<br><br>Q9S795<br>NP_565094<br>NP_001185399<br>ALDH10A8<br>AT1G74920<br>ALDH10A8<br>Chromosome 1<br>501 aa
Atriplex centralasiatica<br>eudicotyledons; core eudicotyledons; Caryophyllales; Amaranthaceae	AAM19157<br>Q8L8I3<br>BADH<br>500aa
Atriplex hortensis<br>(Mountain spinach)<br>eudicotyledons; core eudicotyledons; Caryophyllales; Amaranthaceae	ABF72123<br>Q19QV8<br>(P42575; same gene)<br>500 aa

Organism<br>Lineage<br>Genome assembly<br>Number of genes	ALDH10<br>Protein accession number<br>Gene locus<br>Amino acid sequence length
Atriplex micrantha<br>eudicotyledons; core eudicotyledons; Caryophyllales; Amaranthaceae	ABM97658<br>A2TJX7<br>BADH<br>500 aa
Atriplex prostrata<br>(Spear-leaved orache) (Atriplex triangularis)<br>eudicotyledons; core eudicotyledons; Caryophyllales; Amaranthaceae	AAM08913<br>Q8RX99<br>BADH1<br>(AAP13999)<br>500 aa<br><br>AAM08914<br>Q8RX98<br>BADH2<br>424 aa (fragment)
Atriplex tatarica<br>eudicotyledons; core eudicotyledons; Caryophyllales; Amaranthaceae	ABQ18317<br>A5HMM5<br>BADH<br>500 aa
Avicennia marina (betaine-accumulating mangrove) (Grey mangrove) (*Sceura marina*)<br>eudicotyledons; core eudicotyledons; asterids; lamiids; Lamiales; Acanthaceae; Avicennioideae	BAB18544<br>Q9FRY2<br>Clone 13<br>502 aa<br><br>BAB18543<br>Q9FRY3<br>Clone 2<br>503 aa
Beta vulgaris (sugar beet)<br>eudicotyledons; core eudicotyledons; Caryophyllales; Amaranthaceae<br>NCBI: BioProject PRJNA176558, assembly BwSeq-1<br>9 chromosomes	P28237<br>CAA41377<br>CAA41376<br>Chloroplastic<br>500 aa<br><br>(not reported yet at NCBI)<br>Locus 1572<br>500 aa
Brassica napus (rape)<br>eudicotyledons; core eudicotyledons; rosids; malvids; Brassicales; Brassicaceae; Brassiceae	AAQ55493<br>503 aa
Brassica rapa (field mustard; Turnip)<br>eudicotyledons; core eu dicotyledons; rosids; malvids; Brassicales; Brassicaceae; Brassiceae<br><br>Phytozome: v1.2 annotation is on assembly v1.1.<br>26,374 total loci containing protein-coding transcripts	Bra003781<br>Bra003781<br>501 aa<br><br>Bra019528<br>Bra019528<br>503 aa

**Organism**<br>**Lineage**<br>**Genome assembly**<br>**Number of genes**	**ALDH10**<br>**Protein accession number**<br>**Gene locus**<br>**Amino acid sequence length**
Camellia sinensis (tea)<br>eudicotyledons; core eudicotyledons; asterids; Ericales; Theaceae	AFP19449<br>I7F760<br>505 aa
Capsella rubella<br>Tracheophyta; Spermatophyta; Magnoliophyta; eudicotyledons; core eudicotyledons; rosids; malvids; Brassicales; Brassicaceae; Camelineae<br>Phytozome: initial 22x genome assembly and a preliminary annotation<br>26,521 total loci containing protein-coding transcripts	EOA23841<br>CARUB_v10017058mg<br>503 aa<br><br>EOA35065<br>CARUB_v10020176mg<br>501 aa
Carthamus tinctorius (safflower)<br>eudicotyledons; core eudicotyledons; asterids; campanulids; Asterales; Asteraceae; Carduoideae; Cardueae; Centaureinae	ADW80905<br>G8D1H1<br>510 aa
Chorispora bungeana<br>eudicotyledons; core eudicotyledons; rosids; malvids; Brassicales; Brassicaceae; Chorisporeae	AAV67891<br>Q5Q033<br>502 aa
Chrysanthemum lavandulifolium (Daisy) (*Dendranthema lavandulifolium*)<br>eudicotyledons; core eudicotyledons; asterids; campanulids; Asterales; Asteraceae; Asteroideae; Anthemideae; Artemisiinae	AAY33871<br>Q4U5F2<br>DlBADH1<br>503 aa<br><br>AAY33872<br>Q4U5F1<br>DlBADH2<br>506 aa
Cicer arietinum (chickpea) (garbanzo)<br>Tracheophyta; Spermatophyta; Magnoliophyta; eudicotyledons; core eudicotyledons; rosids; fabids; Fabales; Fabaceae; Papilionoideae; Cicereae	XP_004508822<br>LOC101506136<br>Chromosome Ca7<br>503 aa<br><br>XP_004501961<br>LOC101507930<br>Chromosome Ca5<br>503 aa
Citrus clementina (clementine mandarin)<br>Tracheophyta; Spermatophyta; Magnoliophyta; eudicotyledons; core eudicotyledons; rosids; malvids; Sapindales; Rutaceae<br><br>Phytozome: (clementine1.0) integrates 1.560 M ESTs with homology and ab initio-based gene predictions.<br>9 chromosomes<br>24,533 protein-coding loci	Ciclev10019800m<br>Ciclev10019800m.g<br>505 aa<br><br>Ciclev10019822m<br>Ciclev10019800m.g<br>501 aa<br>(same locus, alternative transcript)

Organism<br>Lineage<br>Genome assembly<br>Number of genes	ALDH10<br>Protein accession number<br>Gene locus<br>Amino acid sequence length
Citrus sinensis (sweet orange)<br>Tracheophyta; Spermatophyta; Magnoliophyta; eudicotyledons; core eudicotyledons; rosids; malvids; Sapindales; Rutaceae<br><br>Phytozome: (v.1) of the assembly is 319 Mb spread over 12,574 scaffolds<br>25,376 protein-coding loci	Located in scaffold_0016_14<br>(non-reported gene)
Corylus heterophylla<br>eudicotyledons; core eudicotyledons; rosids; fabids; Fagales; Betulaceae	ADW80331<br>E9NRZ9<br>503 aa
Cucumis melo (muskmelon)<br>eudicotyledons; core eudicotyledons; rosids; fabids; Cucurbitales; Cucurbitaceae; Benincaseae	AEK81574<br>G1EH68<br>503 aa
Cucumis sativus (cucumber)<br>eudicotyledons; core eudicotyledons; rosids; fabids; Cucurbitales; Cucurbitaceae; Benincaseae<br>Phytozome: Roche/JGI v1 annotation<br>21491 loci containing protein-coding transcripts	XP_004138132<br>LOC101213657<br>Cucsa.197230<br>503 aa
Fragaria vesca subsp. Vesca (woodland strawberry)<br>Tracheophyta; Spermatophyta; Magnoliophyta; eudicotyledons; core eudicotyledons; rosids; fabids; Rosales; Rosaceae; Rosoideae; Potentilleae; Fragariinae<br><br>NCBI BioProject: PRJNA66853, PRJNA60037; assembly: FraVesHawaii_1.0<br>7 chromosomes<br>25,247 genes<br>23,319 proteins<br>Phytozome: includes gene annotation of genome release 1.1.<br>7 chromosomes<br>32,831 loci containing protein-coding transcripts	XP_004299590<br>mrna09492.1-v1.0-hybrid<br>LOC101300913<br>gene09492-v1.0-hybrid<br>505 aa

Organism<br>Lineage<br>Genome assembly<br>Number of genes	ALDH10<br>Protein accession number<br>Gene locus<br>Amino acid sequence length
Glycine max (Soybean) (Glycine hispida)<br>eudicotyledons; core eudicotyledons; rosids; fabids; Fabales; Fabaceae; Papilionoideae; Phaseoleae<br><br>50202 genes (20 chromosomes)<br>44642 proteins<br><br>Phytozome: Soybean Glyma1.0 annotation; 20 chromosomes, with a small additional amount of mostly repetitive sequence in unmapped scaffolds.<br>54,175 protein-coding loci and 73,320 transcripts have been predicted	NP_001234990<br>BADH1<br>B0M1A6<br>Glyma06g19820<br>Chromosome 6<br>503 aa<br><br>NP_001238427<br>B0M1A5<br>ADN03184<br>BADH2<br>Glyma05g01770<br>Chromosome 5<br>503 aa<br><br>XP_003550754<br>LOC100795267<br>Glyma17g10120<br>Chromosome 17<br>315 aa (pseudogene)
Gossypium hirsutum (upland cotton)<br>eudicotyledons; core eudicotyledons; rosids; malvids; Malvales; Malvaceae; Malvoideae	AAR23816<br>503 aa
Gossypium raimondii (cotton)<br>eudicotyledons; core eudicotyledons; rosids; malvids; Malvales; Malvaceae; Malvoideae<br><br>Phytozome: v2.1 annotation release is on genome assembly v2.0<br>13 chromosomes<br>37,505 protein coding genes	Gorai.001G071200.2<br>Gorai.001G071200<br>503 aa<br><br>Gorai.007G047400.1<br>Gorai.007G047400<br>502 aa
Halocnemum strobilaceum<br>eudicotyledons; core eudicotyledons; Caryophyllales; Amaranthaceae	AFB74193<br>H6VX92<br>BADH<br>500 aa
Halostachys caspica<br>eudicotyledons; core eudicotyledons; Caryophyllales; Amaranthaceae	ABO45931<br>A4LAP2<br>500 aa
Haloxylon ammodendron<br>eudicotyledons; core eudicotyledons; Caryophyllales; Amaranthaceae	ACS96437<br>D0E0H5<br>BADH<br>500 aa
Haloxylon persicum<br>eudicotyledons; core eudicotyledons; Caryophyllales; Amaranthaceae	AEW31327<br>G9I1P9<br>BADH<br>500 aa

**Organism**<br>**Lineage**<br>**Genome assembly**<br>**Number of genes**	**ALDH10**<br>**Protein accession number**<br>**Gene locus**<br>**Amino acid sequence length**
Helianthus annuus (common sunflower)<br>eudicotyledons; core eudicotyledons; asterids; campanulids; Asterales; Asteraceae; Asteroideae; Heliantheae alliance; Heliantheae	ACU65243<br>C8CBI9<br>BADH<br>503 aa
Jatropha curcas<br>eudicotyledons; core eudicotyledons; rosids; fabids; Malpighiales; Euphorbiaceae; Crotonoideae; Jatropheae	ABO69575<br>B2BBY6<br>BADH<br>503 aa<br>(AFY98894-521aa, incorrect insertion)
Kalidium foliatum<br>eudicotyledons; core eudicotyledons; Caryophyllales; Amaranthaceae	ABI95806<br>Q06AI9<br>500 aa
Ligusticum sinense<br>eudicotyledons; core eudicotyledons; asterids; campanulids; Apiales; Apiaceae; Apioideae; apioid superclade; Selineae	ADL61811<br>H2KKR8<br>508 aa
Lycium barbarum (Matrimony vine)<br>eudicotyledons; core eudicotyledons; asterids; lamiids; Solanales; Solanaceae; Solanoideae; Lycieae	ACQ99195<br>D2DEK8<br>503 aa
Medicago sativa (alfalfa)<br>eudicotyledons; core eudicotyledons; rosids; fabids; Fabales; Fabaceae; Papilionoideae; Trifolieae	AFS33786<br>J9XXT4<br>BADH<br>505 aa
Medicago truncatula (barrel medic)<br>eudicotyledons; core eudicotyledons; rosids; fabids; Fabales; Fabaceae; Papilionoideae; Trifolieae<br>45000 genes (8chromosomes)<br>46092 proteins	ABE82378<br>XP_003608928<br>G7JNS2<br>(AFK34878)<br>(AFK43263)<br>MTR_4g106510<br>Chromosome 4<br>503 aa<br>(ACJ85836; fragment)<br><br>AFK44601<br>(NC_016409)<br>Chromosome 3<br>503 aa
Mimulus guttatus (spotted monkey flower; Yellow monkey flower)<br>Tracheophyta; Spermatophyta; Magnoliophyta; eudicotyledons; core eudicotyledons; asterids; lamiids; Lamiales; Phrymaceae<br>Phytozome: includes the JGI gene annotation v1.1 of assembly v1.0<br>26718 loci containing protein-coding genes	mgv1a004929m<br>mgv1a004929m.g<br>504 aa
Morus alba var. Multicaulis (mulberry)<br>Tracheophyta; Spermatophyta; Magnoliophyta; eudicotyledons; core eudicotyledons; rosids; fabids; Rosales; Moraceae	AGG82698<br>M4NDU5<br>501 aa

**Organism**<br>**Lineage**<br>**Genome assembly**<br>**Number of genes**	**ALDH10**<br>**Protein accession number**<br>**Gene locus**<br>**Amino acid sequence length**
Panax ginseng (Korean ginseng)<br>eudicotyledons; core eudicotyledons; asterids; campanulids; Apiales; Araliaceae	AAQ76705<br>Q6JSK3<br>BADH1<br>503 aa
Phaseolus vulgaris (Kidney bean; French bean)<br>eudicotyledons; core eudicotyledons; rosids; fabids; Fabales; Fabaceae; Papilionoideae; Phaseoleae<br><br>Phytozome: release of V1.0, the first chromosome scale version<br>11 chromosomes	Phvul.009G182300.1<br>Phvul.009G182300<br>Chromosome 9<br>503 aa<br><br>Phvul.003G196700.1<br>Phvul.003G196700<br>Chromosome 3<br>503 aa
Pisum sativum (pea)<br>eudicotyledons; core eudicotyledons; rosids; fabids; Fabales; Fabaceae; Papilionoideae; Fabeae	CAC48393<br>Q93YB2<br>(PDB: 3IWJ)<br>AMADH2<br>503 aa<br><br>CAC48392<br>(PDB: 3IWK)<br>AMADH1<br>503 aa
Populus euphratica (Euphrates poplar)<br>eudicotyledons; core eudicotyledons; rosids; fabids; Malpighiales; Salicaceae; Saliceae	AFA53117<br>H6V966<br>BADH2<br>503 aa<br><br>AFA53116<br>H6V965<br>BADH1<br>503 aa
Populus trichocarpa (*Populus balsamifera* subsp. trichocarpa) (black cottonwood tree; Western balsam poplar))<br>eudicotyledons; core eudicotyledons; rosids; fabids; Malpighiales; Salicaceae; Saliceae<br><br>JGI v3.0 gene annotation of assembly v3<br>41335 loci containing protein-coding transcripts<br>73013 protein-coding trancripts<br><br>45555 protein-coding genes (19 chromosomes)	XP_002322147<br>(B9IEL4)<br>Potri.015G070600<br>(POPTRDRAFT_666405)<br>Chromosome LGXV<br>503 aa<br><br>XP_002318630<br>(B9I351)<br>Potri.012G075600<br>(POPTRDRAFT_661953)<br>Chromosome LGXII<br>503 aa

Organism<br>Lineage<br>Genome assembly<br>Number of genes	ALDH10<br>Protein accession number<br>Gene locus<br>Amino acid sequence length
Prunus persica (peach)<br>Tracheophyta; Spermatophyta; Magnoliophyta; eudicotyledons; core eudicotyledons; rosids; fabids; Rosales; Rosaceae; Maloideae; Amygdaleae	EMJ18033<br>M5X943<br>PRUPE_ppa022568mg<br>503 aa<br><br>EMJ11127<br>M5WBJ6<br>PRUPE_ppa004563mg<br>503 aa
Pyrus betulifolia (birch-leaf pear)<br>eudicotyledons; core eudicotyledons; rosids; fabids; Rosales; Rosaceae; Amygdaloideae; Maleae	AER10508<br>G8EWJ4<br>503 aa
Ricinus communis (Castor bean)<br>eudicotyledons; core eudicotyledons; rosids; fabids; Malpighiales; Euphorbiaceae; Acalyphoideae; Acalypheae<br><br>Phytozome: includes TIGR/JCVI release v0.1<br>31221 protein-coding transcripts	XP_002511463<br>B9R8Y8<br>RCOM_1512620<br>30147.t000284<br>503 aa
Sesuvium portulacastrum (Shoreline sea purslane) (Portulaca portulacastrum)<br>eudicotyledons; core eudicotyledons; Caryophyllales; Aizoaceae	AEK98521<br>G1FG21<br>500aa
Solanum lycopersicum (tomato) (Lycopersicon esculentum)<br>eudicotyledons; core eudicotyledons; asterids; lamiids; Solanales; Solanaceae; Solanoideae; Solaneae<br>NCBI BioProject: PRJNA66163, assembly SL2.40<br>12 chromosomes<br>27,466 genes<br>Phytozome: ITAG2.3 made by International Tomato Annotation Group (ITAG) on assembly ITAG2.3<br>12 chromosomes<br>34,727 loci containing protein-coding transcripts	AAX73303<br>Q56R04<br>(PDB: 4I8Q; 4I9B)<br>Chromosome 6<br>AMADH1<br>Solyc06g071290.2<br>504 aa<br><br>NP_001234235<br>B6ECN9<br>Chromosome 3<br>AMADH2<br>Solyc03g113800.2<br>505 aa
Solanum torvum<br>eudicotyledons; core eudicotyledons; asterids; lamiids; Solanales; Solanaceae; Solanoideae; Solaneae	AFA37976<br>H6V8T9<br>BADH<br>505 aa
Solanum tuberosum (Potato) **(SOLTU)**<br>eudicotyledons; core eudicotyledons; asterids; lamiids; Solanales; Solanaceae; Solanoideae; Solaneae<br><br>Phytozome: Solanum tuberosum Group Phureja DM1-3 516R44 (CIP801092) Genome Annotation v3.4 mapped to pseudomolecule sequence<br>12 chromosomes<br>35,119 loci containing protein-coding transcripts	PGSC0003DMP400055759<br>PGSC0003DMT400083025<br>PGSC0003DMG400033028<br>504 aa<br><br>PGSC0003DMP400042549<br>PGSC0003DMT400063205<br>PGSC0003DMG400024582<br>505 aa

Organism<br>Lineage<br>Genome assembly<br>Number of genes	ALDH10<br>Protein accession number<br>Gene locus<br>Amino acid sequence length
Spinacia oleracea (spinach)<br>Tracheophyta; Spermatophyta; Magnoliophyta; eudicotyledons; core eudicotyledons; Caryophyllales; Amaranthaceae; Chenopodioideae; Anserineae	P17202<br>(PDB: 4A0M)<br>chloroplastic<br>497 aa
Suaeda liaotungensis<br>eudicotyledons; core eudicotyledons; Caryophyllales; Amaranthaceae	AAL33906<br>Q8W5A1<br>BADH<br>501aa
Suaeda maritima (Annual sea blite) (Suaeda spicata)<br>eudicotyledons; core eudicotyledons; Caryophyllales; Amaranthaceae	AFW04226<br>K7T3P2<br>501 aa
Suaeda salsa (Seepweed) (Chenopodium salsum)<br>eudicotyledons; core eudicotyledons; Caryophyllales; Amaranthaceae	ABG23669<br>Q155V4<br>BADH<br>501 aa
Theobroma cacao cultivar="Matina 1-6" (cacao) (cocoa)<br>Tracheophyta; Spermatophyta; Magnoliophyta; eudicotyledons; core eudicotyledons; rosids; malvids; Malvales; Malvaceae; Byttnerioideae<br><br>Phytozome: V1.1 release<br>29,452 total loci containing protein-coding transcripts	EOY20585<br>Domain 1<br>TCM_011968<br>Chromosome 3<br>503 aa<br><br>EOY20585<br>Domain 2<br>TCM_011968<br>Chromosome 3<br>511 aa<br>Comprise two adjacent ALDH10 genes

**Organism**<br>**Lineage**<br>**Genome assembly**<br>**Number of genes**	**ALDH10**<br>**Protein accession number**<br>**Gene locus**<br>**Amino acid sequence length**
Vitis vinifera (wine grape)<br>eudicotyledons; core eudicotyledons; rosids; Vitales; Vitaceae<br><br>NCBI BioProject: PRJNA34679<br>19 chromosomes<br>24,508 genes<br>Phytozome: 12X March 2010 release of the draft genome and annotation of Vitis vinifera by the French-Italian Public Consortium for Grapevine Genome Characterization<br>19 chromosomes<br>26346 loci containing protein-coding transcripts	XP_003634296<br>LOC100251899<br>Chromosome 17<br>497 aa<br>(CBI15092) fragment<br><br>XP_002283690<br>D7SHY3<br>GSVIVT01007829001<br>LOC100246770<br>GSVIVT01007829001<br>Chromosome 17<br>503 aa<br><br>XP_002281984<br>GSVIVG01032588001<br>LOC100250859<br>GSVIVG01032588001<br>Chromosome 14<br>499 aa
**Fungi**	
**Ascomycota**	
Schizosaccharomyces pombe (fission yeast)<br>Fungi; Dikarya; Ascomycota; Taphrinomycotina; Schizosaccharomycetes; Schizosaccharomycetales; Schizosaccharomycetaceae<br>Bioproject: PRJNA127, PRJNA13836, PRJNA20755<br>3 chromosomes<br>5133 protein coding genes	O59808<br>NP_588102<br>SPCC550.10<br>Chromosome III<br>500 aa
**Protist**	
**Alveolata**	
Perkinsus marinus ATCC 50983<br>Eukaryota; Alveolata; Perkinsea; Perkinsida; Perkinsidae<br>Bioproject: PRJNA46451, PRJNA12737<br>23654 protein coding genes	XP_002784436<br>Pmar_PMAR003695<br>394aa (fragment)<br>incomplete on both ends
**Cryptophyta**	
Guillardia theta CCMP2712<br>Eukaryota; Cryptophyta; Pyrenomonadales; Geminigeraceae<br>Bioproject: PRJNA223305, PRJNA53577<br>24822 protein coding genes	EKX35999<br>GUITHDRAFT_117911<br>542 aa
**Stramenopiles**	
Ectocarpus siliculosus (brown alga)<br>Eukaryota; Stramenopiles; PX clade; Phaeophyceae; Ectocarpales; Ectocarpaceae<br>Bioproject: PRJEA42625<br>4005 protein coding genes	CBN79171<br>Esi_0010_0069<br>517 aa

**Organism**<br>**Lineage**<br>**Genome assembly**<br>**Number of genes**	**ALDH10**<br>**Protein accession number**<br>**Gene locus**<br>**Amino acid sequence length**
Phytophthora sojae<br>Eukaryota; Stramenopiles; Oomycetes; Peronosporales<br>Bioproject: PRJNA17989<br>26489 protein coding genes	EGZ28599<br>G4YMH3<br>PHYSODRAFT_248120<br>473 aa<br><br>EGZ28472<br>G4YIQ2<br>PHYSODRAFT_284276<br>418 aa
**Bacteria**	
**Alphaproteobacteria**	
Rhizobium leguminosarum bv. trifolii WSM2297<br>Bacteria; Proteobacteria; Alphaproteobacteria; Rhizobiales; Rhizobiaceae; Rhizobium/Agrobacterium group	WP_003575619<br>EJC83393<br>ZP_18315655<br>Rleg4DRAFT_5155<br>496 aa
Rhizobium sp. CCGE 510<br>Bacteria; Proteobacteria; Alphaproteobacteria; Rhizobiales; Rhizobiaceae; Rhizobium/Agrobacterium group	EJT02404<br>ZP_10838419<br>J6DKY5<br>RCCGE510_27926<br>plasmid="pRspCCGE510d"<br>496 aa
**Betaproteobacteria**	
Burkholderia ambifaria MC40-6<br>Betaproteobacteria; Burkholderiales; Burkholderiaceae; Burkholderia<br>6784 genes	ZP_01555907<br>YP_001809915<br>BamMC406_3226<br>Chromosome 2<br>493 aa
Burkholderia multivorans ATCC 17616<br>Betaproteobacteria; Burkholderiales; Burkholderiaceae; Burkholderia<br>6373 genes	ZP_01568328<br>YP_001585229<br>Bmul_5267<br>Chromosome 2<br>493 aa
Burkholderia pseudomallei 305<br>Betaproteobacteria; Burkholderiales; Burkholderiaceae; Burkholderia	ZP_01765086<br>BURPS305_6395<br>482 aa
Burkholderia sp. 383<br>Burkholderia cepacia R18194<br>Betaproteobacteria; Burkholderiales; Burkholderiaceae; Burkholderia<br>7824 genes	YP_366413<br>Bcep18194_C6720<br>Chromosome 3<br>490 aa
**Gammaproteobacteria**	
Colwellia psychrerythraea 34H **(COLPS)**<br>5054 genes (chromosome)	YP_266864<br>CPS_0096<br>491 aa

**Organism**<br>**Lineage**<br>**Genome assembly**<br>**Number of genes**	**ALDH10**<br>**Protein accession number**<br>**Gene locus**<br>**Amino acid sequence length**
Pseudomonas fluorescens Pf0-1<br>Gammaproteobacteria; Pseudomonadales; Pseudomonadaceae; Pseudomonas<br>5833 genes (chromosome)	YP_350198<br>Pfl01_4470<br>483 aa
Pseudomonas protegens Pf-5 **(PSEF5)**<br>Gammaproteobacteria; Pseudomonadales; Pseudomonadaceae; Pseudomonas<br>6233 genes (chromosome)	YP_261892<br>PFL_4811<br>482 aa<br><br>YP_260018<br>PFL_2912<br>476 aa
Pseudomonas putida W619 **(PSEPW)**<br>5309 genes (chromosome)	ZP_01639992<br>YP_001751324<br>PputW619_4475<br>476 aa
