# Supplementary material for: Exploring the evolutionary route of the acquisition of betaine aldehyde dehydrogenase activity by plant ALDH10 enzymes: implications for the synthesis of the osmoprotectant glycine betaine
Source: BMC Plant Biol. 2014 May 29;14:149. doi: 10.1186/1471-2229-14-149 (PMC4046141; doi:10.1186/1471-2229-14-149)
Supplement: Additional file 2: Figure S1 — Interactions of the residue at position equivalent to A441 of SoBADH. Table S2. Distances of the side-chain atoms of the residue at position 441 to their closest neighbors. [file 1471-2229-14-149-S2.pdf]

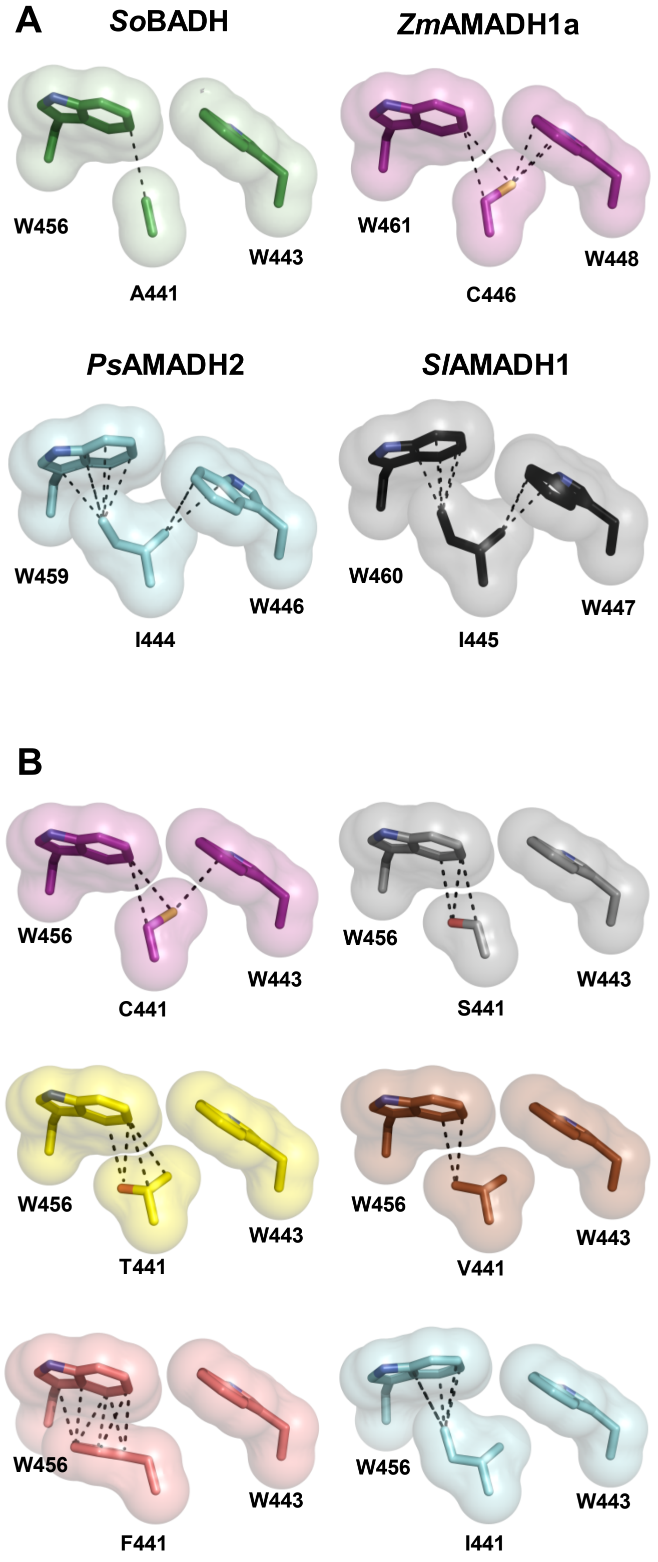

Figure S1 Interactions of the residue at position equivalent to Ala441 of *So*BADH. **(A)** Molecular surface representations showing the side-chains of residues at position 441, 443 and 456 (*So*BADH numbering) in the known crystal structures of plant ALDH10 enzymes. (**B**) Molecular surface representations of the same residues in the minimized models of the *in silico So*BADH mutants in which A441 was changed. Side-chains are shown as sticks with oxygen atoms in red, nitrogen in blue, and sulfur in yellow. Carbon atoms are colored as shown. The following PDB codes were used: *So*BADH, 4A0M; *Ps*AMADH2, 3IWJ; *Zm*AMADH1a, 4I8P; *Sl*AMADH1, 4I9B. The figure was generated using PyMOL (www.pymol.org).

Table S2. Distances of the side-chain atoms of residue at position 441 to their closets neighbor

Enzyme (residue)	Atoms	Distance (Å)
*So*BADH (A441)	CB A441-CZ3 W456	3.71
*Zm*AMADH1 (C446)	CB C446-CZ3 W461	3.90
	SG C446-CZ3 W461	3.94
	SG C446-CZ2 W448	3.62
	SG C446-CE2 W448	3.52
	SG C446-NE1 W448	3.76
*Ps*AMADH2[b] (I444)	CD1 I444-CG W459	3.90
	CD1 I444-CD2 W459	3.39
	CD1 I444-CE2 W459	3.54
	CD1 I444-CZ2 W459	3.85
	CD1 I444-CZ3 W459	3.84
	CD1 I444-CE3 W459	3.55
	CG2 I444-CH2 W446	3.74
	CG2 I444-CZ2 W446	3.59
	CG2 I444-CE2 W446	3.81
*Sl*AMADH (I445)	CD1 I445-CH2 W460	3.81
	CD1 I445-CD2 W460	3.51
	CD1 I445-CE2 W460	3.56
	CD1 I445-CZ2 W460	3.74
	CD1 I445-CZ3 W460	3.76
	CD1 I445-CE3 W460	3.62
	CG2 I445-CH2 W447	3.81
	CG2 I445-CZ2 W447	3.54
	CG2 I445-CE2 W447	3.67
*So*BADH A441C mutant	CB C441-CZ3 W456	3.67
	SG C441-CZ3 W456	3.94
	SG C441-CE2 W443	3.74
*So*BADH A441S mutant	CB S441-CZ3 W456	3.67
	OG S441-CZ3 W456	3.56
	OG S441-CE3 W456	3.50
*So*BADH A441T mutant	CB T441-CZ3 W456	3.69
	CG2 T441-CZ3 W456	3.72
	OG1 T441-CZ3 W456	3.34
	OG1 T441-CE3 W456	3.30
*So*BADH A441V mutant	CG2 V441-CZ3 W456	3.73
	CG2 V441-CE3 W456	3.70
*So*BADH A441F mutant	CG F441-CZ3 W456	3.60
	CG F441-CE3 W456	3.56
	CD1 F441-CE3 W456	3.70
	CE1 F441-CE3 W456	3.67
	CZ F441-CE3 W456	3.48
	CZ F441-CD2 W456	3.70
	CZ F441-CG W456	3.74
	CD2 F441-CZ3 W456	3.59
	CD2 F441-CE3 W456	3.36
	CE2 F441-CE3 W456	3.32
	CE2 F441-CD2 W456	3.40
	CE2 F441-CG W456	3.71
	CZ F441-CE3 W456	3.48
	CZ F441-CD2 W456	3.70
	CZ F441-CG W456	3.74
*So*BADH A441I mutant	CD1 I441-CZ3 W456	3.41
	CD1 I441-CH2 W456	3.86
	CD1 I441-CE3 W456	3.17
	CD1 I441-CD2 W456	3.41
	CD1 I441-CE2 W456	3.85

[a]The distances given are the average of those observed in the two monomers of each crystal structure or model. The cutoff was made at 4.0 Å. [b]The crystal structure of the *Ps*AMDH2 enzyme is very similar in this region to that of *Ps*AMDH1, which is not included here for this reason. The PDB codes of the crystal structures are: *So*BADH, 4A0M; *Zm*AMADH1a, 4I8P; *Ps*AMADH2, 3IWJ; *Sl*AMADH1, 4I9B.
